# The chloroplast genomes of *Bryopsis plumosa* and *Tydemania expeditiones* (Bryopsidales, Chlorophyta): compact genomes and genes of bacterial origin

**DOI:** 10.1186/s12864-015-1418-3

**Published:** 2015-03-17

**Authors:** Frederik Leliaert, Juan M Lopez-Bautista

**Affiliations:** Department of Biological Sciences, The University of Alabama, Tuscaloosa, AL USA; Department of Biology, Marine Biology Research Group, Ghent University, Krijgslaan 281-S8, Ghent, 9000 Belgium

**Keywords:** Horizontal gene transfer, Introns, Mobile elements, Phylogenomics, Plastid genome, Viridiplantae

## Abstract

**Background:**

Species of Bryopsidales form ecologically important components of seaweed communities worldwide. These siphonous macroalgae are composed of a single giant tubular cell containing millions of nuclei and chloroplasts, and harbor diverse bacterial communities. Little is known about the diversity of chloroplast genomes (cpDNAs) in this group, and about the possible consequences of intracellular bacteria on genome composition of the host. We present the complete cpDNAs of *Bryopsis plumosa* and *Tydemania expeditiones*, as well as a re-annotated cpDNA of *B. hypnoides*, which was shown to contain a higher number of genes than originally published. Chloroplast genomic data were also used to evaluate phylogenetic hypotheses in the Chlorophyta, such as monophyly of the Ulvophyceae (the class in which the order Bryopsidales is currently classified).

**Results:**

Both DNAs are circular and lack a large inverted repeat. The cpDNA of *B. plumosa* is 106,859 bp long and contains 115 unique genes. A 13 kb region was identified with several freestanding open reading frames (ORFs) of putative bacterial origin, including a large ORF (>8 kb) closely related to bacterial *rhs*-family genes. The cpDNA of *T. expeditiones* is 105,200 bp long and contains 125 unique genes. As in *B. plumosa*, several regions were identified with ORFs of possible bacterial origin, including genes involved in mobile functions (transposases, integrases, phage/plasmid DNA primases), and ORFs showing close similarity with bacterial DNA methyltransferases. The cpDNA of *B. hypnoides* differs from that of *B. plumosa* mainly in the presence of long intergenic spacers, and a large tRNA region. Chloroplast phylogenomic analyses were largely inconclusive with respect to monophyly of the Ulvophyceae, and the relationship of the Bryopsidales within the Chlorophyta.

**Conclusions:**

The cpDNAs of *B. plumosa* and *T. expeditiones* are amongst the smallest and most gene dense chloroplast genomes in the core Chlorophyta. The presence of bacterial genes, including genes typically found in mobile elements, suggest that these have been acquired through horizontal gene transfer, which may have been facilitated by the occurrence of obligate intracellular bacteria in these siphonous algae.

**Electronic supplementary material:**

The online version of this article (doi:10.1186/s12864-015-1418-3) contains supplementary material, which is available to authorized users.

## Background

The circa 70 chloroplast genomes (cpDNAs) of green algae sequenced to date have revealed a remarkable array of genomic architectures, including a wide range of genome sizes, gene repertoires and arrangements, and nucleotide compositions [1-3]. Many green algal cpDNAs have a quadripartite structure also found in land plants, characterized by the presence of two copies of a large inverted repeat sequence separating a small and a large single-copy region. Although this architecture is believed to be ancestral in the green algae, many species (including several Chlorophyta) do not have a quadripartite structure [4-10].

**Figure 1:**
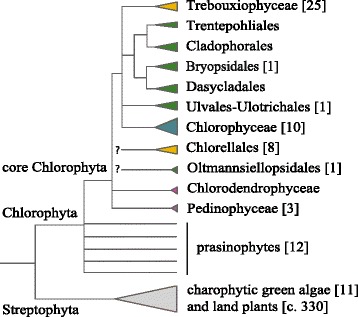
**Phylogenetic relationships among the main clades of green algae, focusing on the core Chlorophyta.** The tree is a composite of accepted relationships based on molecular phylogenetic evidence from different studies [17-19]. Uncertain or conflicting relationships are indicated by polytomies or question marks. Numbers in square brackets indicate the number of (nearly) complete cpDNAs available to date. Clades currently classified as Ulvophyceae are in green, and Trebouxiophyceae are in yellow.

Complete cpDNA sequences are only available for three species of Ulvophyceae (Figure 1), including the marine flagellate *Oltmannsiellopsis viridis* (Oltmannsiellopsidales), the freshwater microfilamentous *Pseudendoclonium akinetum* (Ulotrichales), and the marine siphonous species *Bryopsis hypnoides* (Bryopsidales) [11-13]. The cpDNAs of *Oltmannsiellopsis* (151.9 kb) and *Pseudendoclonium* (195.8 kb) share the quadripartite architecture, and a similar large complement of genes (104 and 105 genes, respectively), but differ in size due to different intron numbers (five in *Oltmannsiellopsis* vs 27 in *Pseudendoclonium*). The 153.4 kb cpDNA of *B. hypnoides* differs in several aspects from the two other species of Ulvophyceae. It does not feature a quadripartite architecture, and it includes 10 tRNA genes that are not present in any other green algal cpDNA. Another peculiarity of the *B. hypnoides* cpDNA is the presence of multimeric forms of the cpDNA, including monomers, dimers, trimers, tetramers, and higher-order multimers, which were detected by pulsed-field gel electrophoresis and Southern blot methods [13]. 111 genes were reported in the cpDNA of *B. hypnoides* but our preliminary analysis indicated a higher number of genes, as also suggested by others [1]. Apart from completely sequenced cpDNAs, partial chloroplast genome data in the Bryopsidales has long been available for *Codium fragile* [14] and *Caulerpa sertularoides* [15] through Southern hybridization analysis of restriction fragments. In addition partial DNA sequence data is available for *Caulerpa filiformis* [16].

The relationships within the core Chlorophyta have recently been evaluated based on multigene datasets [17-19], which is relevant in the light of comparative genomics. Although monophyly of the Ulvophyceae was supported in a 10-gene phylogeny (eight nuclear and two chloroplast encoded genes) [17], chloroplast (cp) multigene phylogenetic analyses generally failed to recover the Ulvophyceae as a clade. For example, phylogenetic analysis of 23 cp genes recovered *Caulerpa* (Bryopsidales) as more closely related to *Chlorella* (Trebouxiophyceae) than to the other two ulvophycean taxa in the phylogeny (*Oltmannsiellopsis* and *Pseudendoclonium*) [16], and a phylogeny inferred from 42 cp genes indicated a relationship between *Bryopsis* and Chlorophyceae [13]. Phylogenomic analyses with increased taxon sampling (53 taxa, 7 cp genes + 18S; and 38 taxa, 53 cp genes) suggested a relationship between *Oltmannsiellopsis* and *Tetraselmis* (Chlorodendrophyceae), and like in the two previous studies did not provide support for a monophyletic Ulvophyceae [18]. In addition, cp multigene phylogenies have rejected monophyly of the Trebouxiophyceae, which was shown to fall apart in at least two main clades [18,19]. In general, relationships among the major lineages of core Chlorophyta remain poorly supported. As illustrated in Figure 1, complete data on chloroplast genomes is scarce in the Ulvophyceae compared to other green algal lineages, with data lacking for several important clades, including the Cladophorales, Dasycladales, and Trentepohliales.

This paper focusses on the siphonous marine green algae *Bryopsis* and *Tydemania*, two members of the order Bryopsidales [20]. Species in this order form thalli that are composed of a single, giant cell, containing millions of nuclei, chloroplasts and mitochondria that move about freely by cytoplasmic streaming. *Bryopsis* includes more than 20 species, all characterized by morphologically simple feather-like thalli, mostly only a few cm tall. The genus is distributed worldwide in tropical to temperate seas. *Tydemania* forms more complex and larger thalli composed of whorls of siphons arising from a branched main axis. The genus includes a single species, *T. expeditiones*, that is found in the marine tropical Indo-West Pacific. The order Bryopsidales includes two main clades, Bryopsidineae and Halimedineae. *Bryopsis* is a member of the Bryopsidineae, which also includes *Derbesia* and *Codium*, and some other, less known genera. *Tydemania* is a member of the Halimedineae, which also includes some of the better known siphonous green seaweeds such as *Caulerpa* and *Halimeda*. The placement of the Bryopsidales in the class Ulvophyceae has been implied by 18S data and nuclear multi-gene phylogenetic analysis [17,21], but as mentioned above, analyses based on cp genes have casted doubt on the affinity of siphonous green algae with other clades of Ulvophyceae [13,16,18].

In this paper we report on the complete cpDNAs of *Bryopsis plumosa* and *Tydemania expeditiones*, and a re-annotated cpDNA of *B. hypnoides*. We compared these genomes with previously published cpDNAs in the core Chlorophyta to gain a better understanding of the evolution of chloroplast genomes in this group, and to assess relationships among the main clades of core Chlorophyta.

## Results and discussion

### cpDNA assembly, size and organization

For both *Bryopsis plumosa* and *Tydemania expeditiones*, assemblies yielded a single cpDNA sequence, which could be closed into a circle by an overlap of more than 300 base pairs. Both assemblies showed high overall read coverage, and as a result did not contain any ambiguous regions (Additional file 1). For *B. plumosa*, 467,353 reads of a total of 7.2 million mapped to the cpDNA with a mean coverage of 430× (min. 4×, max. 871×). The 4× coverage was 3 bp long and situated in the 3’-end of the *cysT* gene, and was spanned by paired-end reads. For *T. expeditiones*, 7.1 million reads of a total of 14.7 million mapped to the cpDNA with a mean coverage of 6,667× (min. 749×, max. 12,274×).

**Figure 2:**
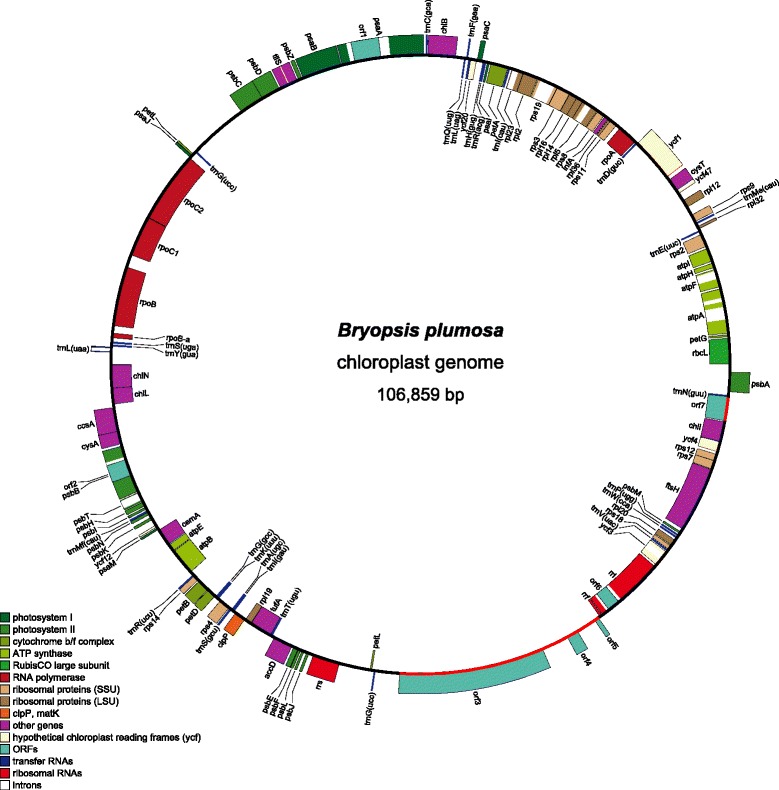
**Gene map of the chloroplast genome of** ***Bryopsis plumosa*** **.** The 106,859 bp genome contains 115 unique genes, including three ribosomal RNA genes, 26 transfer RNA genes, and 86 protein coding genes. Genes shown on the outside of the circle are transcribed counterclockwise. Annotated genes are colored according to the functional categories shown in the legend bottom left. The red arcs indicate gene regions of putative bacterial origin.

**Figure 3:**
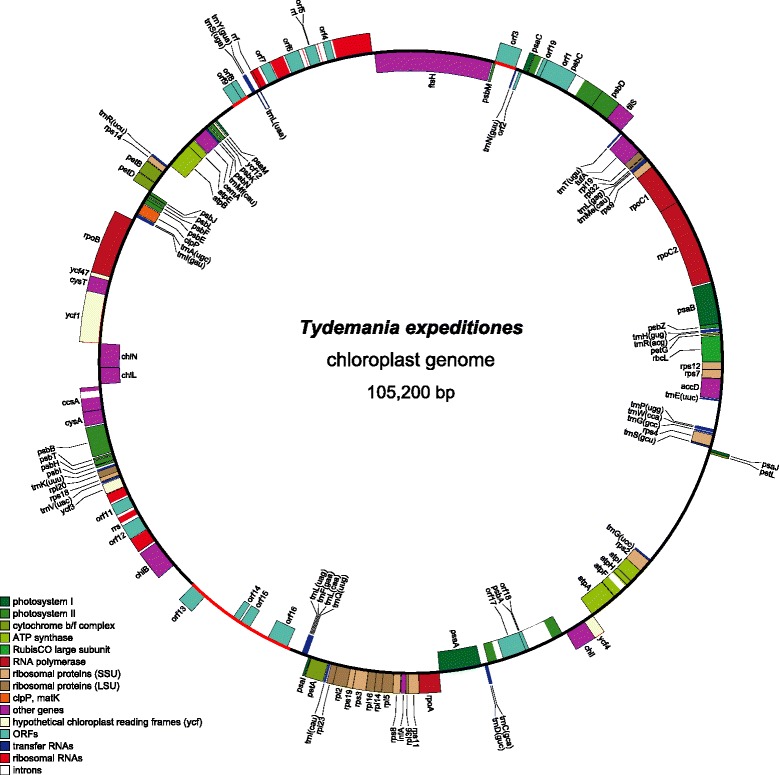
**Gene map of the chloroplast genome of** ***Tydemania expeditiones*** **.** The 105,200 bp genome contains 125 unique genes, including three ribosomal RNA genes, 28 transfer RNA genes, and 94 protein coding genes. Genes shown on the outside of the circle are transcribed counterclockwise. Annotated genes are colored according to the functional categories shown in the legend bottom left. The red arcs indicate gene regions of putative bacterial origin.

The circular cpDNAs of *B. plumosa* (Figure 2) and *T. expeditiones* (Figure 3) consist of 106,859 bp and 105,200 bp, respectively. This is smaller than most published cpDNAs of free-living species of core Chlorophyta, but similar to those found in *Pedinomonas minor* (98 kb), *Marvania geminata* (108 kb), *Pseudochloris wilhelmii* (110 kb), and *Planctonema lauterbornii* (114 kb) [9,19,22]. Most prasinophytes have even smaller cpDNAs, ranging between 64-86 kb, with the exception of *Nephroselmis*, which has larger chloroplast genomes (125 and 201 kb) [3]. Unexpectedly, the cpDNA of *B. plumosa* is much smaller than the 153,429 bp cpDNA of *B. hypnoides*. This size difference is mainly a result of large intergenic spacers (up to 20,875 bp in length), totaling 59,842 bp or 38.9% of the cpDNA in *B. hypnoides* (Additional file 2). Intergenic spacers in *B. plumosa* and *T. expeditiones* account for only 20.3% and 16.3% of the total cpDNA. Nevertheless, some long intergenic spacer regions are present in both genomes. In *B. plumosa*, the largest intergenic spacer was 3,307 bp long. This region contains a few ORFs (>300 bp), none of which showed significant homology to known proteins (blastp E-values > 0.1). In *T. expeditiones*, a large spacer region (6,583 bp) contains ten ORFs (>300 bp), none with significant homology to known proteins (blastp E-values > 1). GC content of the two genomes falls within the limits of other ulvophycean and green algal cpDNAs (Table 1) [1,2].

**Table 1:** **Summary of the** ***Bryopsis plumosa*** **and** ***Tydemania expeditiones*** **cpDNAs and comparison with other ulvophycean cpDNAs**

**Species**	**cpDNA size (kb)**	**GC content (%)**	**Gene number (unique genes)**	**Protein coding genes/ORFs** ^**d**^	**rRNA genes**	**tRNA genes**	**% Coding** ^**b**^	**Intergenic space (%)**	**Intron (%)** ^**c**^	**Intron (%)** ^**b**^	**Intron number**	**Quadripartite structure**	**Reference**
*B. plumosa*	106.9	30.8	115	79/7	3	26	74.4	20.1	5.5	8.3	13	-	This study
*T. expeditiones*	105.2	32.8	125	77/17	3	28	77.5	16.3	6.3	11.2	11	-	This study
*B. hypnoides* ^a^	153.4	33.1	131	78/11	3	39	55.9	38.9	5.2	7.1	11	-	[13]^a^
*P. akinetum*	195.8	31.5	135	74/29	3	29	53.9	37.5	8.5	12.3	28	+	[12]
*O. viridis*	151.9	40.5	112	75/8	3	26	57.9	39.5	2.5	6.8	10	+	[11]

^a^Re-annotated in this study (see Additional file 2).

^b^Including intronic open reading frames (ORFs).

^c^Excluding intronic ORFs.

^d^Only those ORFs are included, which were found to have a significant blastp result (E value < 1e-04).

The *B. plumosa* and *T. expeditiones* cpDNAs both lack a large inverted repeat, similar to the situation found in *B. hypnoides* [13]. The lack of a quadripartite architecture in Bryopsidales had earlier been implied based on Southern hybridization analysis of restriction fragments in *Codium fragile* and *Caulerpa sertularoides* [14,15]. Most plastid genomes have a quadripartite structure where a set of large inverted repeats (typically containing the rRNA genes and some other genes) divides the genome into two single-copy regions. Although the quadripartite structure is believed to be ancestral in Viridiplantae, loss of the inverted repeat has occurred multiple times in different green algal lineages [3,7-9,23,24].

### Gene content

**Figure 4:**
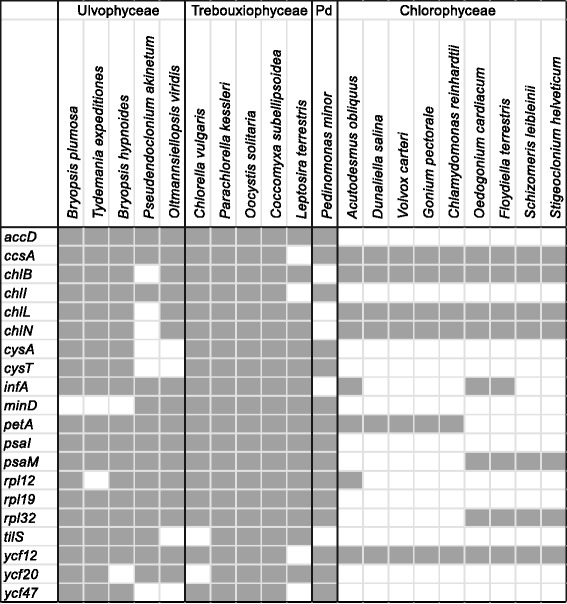
**Comparison of protein coding genes content among core Chlorophyta.** Pd = Pedinophyceae. 80 genes that are shared among the 20 cpDNAs are not included: *atpA*, *B*, *E*, *F*, *H*, *cemA*, *clpP*, *ftsH*, *petB*, *D*, *G*, *L*, *psaA*, *B*, *C*, *J*, *psbA*, *B*, *C*, *D*, *E*, *F*, *H*, *I*, *J*, *K*, *L*, *M*, *N*, *T*, *Z*, *rbcL*, *rpl2*, *5*, *14*, *16*, *20*, *23*, *36*, *rps2*, *3*, *4*, *7*, *8*, *9*, *11*, *12*, *14*, *18*, *19*, *tufA*, *ycf1*, ycf3, *ycf4*, *rpoA*, *B*, *C1*, *C2*, *rrf*, *rrl*, *rrs*, and 20 tRNA genes (*trnA*, *C*, *D*, *E*, *F*, *G*, *H*, *I*, *K*, *L*, *M*, *N*, *P*, *Q*, *R*, *S*, *T*, *V*, *W*, *Y*). *ftsH* and *ycf1* are present in *C. reinhardtii* as ORF2971 and ORF1995, respectively, and *ycf1* is present in *C. vulgaris* as ORF819 ([91,92] (supplementary Table II)). *ycf47* is present in *C. vulgaris* as ORF70 (determined by blastx). Data sources: *B. hypnoides* [13], *P. akinetum* [12], *O. viridis* [11], *C. vulgaris* [6], *P. kessleri*, *O. solitaria*, *P. minor* [22], *C. subellipsoidea* [93], *L. terrestris* [8], *A. obliquus* [94], *D. salina* [95], *V. carteri* [96], *G. pectorale* [97], *C. reinhardtii* [98], *O. cardiacum* [99], *F. terrestris* [7], *S. leibleinii* [4], *S. helveticum* [5].

The *B. plumosa* cpDNA contains 115 unique genes, including 86 protein coding genes, 26 tRNA genes, and three rRNA genes (Tables 1, 2, Figure 4). In addition to expected, conserved plastid protein coding genes, several freestanding ORFs were identified that did not show any relationship with known plastid genes, but instead showed close similarity with bacterial genes (Table 3). The large *orf3* (8,031 bp) showed close similarity to bacterial *rhs*-family proteins. A similar but slightly smaller ORF (7,173 bp, interrupted by a 267 bp intron) was also found in the re-annotated cpDNA of *B. hypnoides* (Additional file 2). *Rhs* genes are a family of composite genes that occur in several Gram-negative bacteria, including some Cyanobacteria. Their broad phylogenetic distribution has been explained by a complex evolutionary history of horizontal transfers and independent gene losses [25]. Despite their wide occurrence, the function of *rhs* genes remains elusive [26]. Other ORFs of putative bacterial origin include a gene encoding DNA polymerase with maturase-specific domain (*orf7*), and two ORFs (*orf4* and *orf5*) showing close similarity to uncharacterized bacterial proteins. *Orf3*, *orf4* and *orf5* are grouped in a 13 kb region of the cpDNA, while *orf7* is situated elsewhere (Figure 2, indicated in red).

**Table 2:** **Comparison of tRNA genes in the two** ***Bryopsis*** **species,** ***Tydemania expeditiones*** **and two other species of Ulvophyceae (** ***Oltmannsiellopsis viridis*** **and** ***Pseudendoclonium akinetum*** **)**

	***B. hyp***	***B. plu***	***T. exp***	***P. aki***	***O. vir***
*trnA*(agc)	1*				
*trnA*(cgc)	1*				
*trnA*(ugc)	1,1*	1	1	2	2
*trnC*(gca)	1,1*	1	1	1	1
*trnD*(guc)	1,1*	1	1	1	1
*trnE*(cuc)	1*				
*trnE*(uuc)	1	1	1	1	1
*trnF*(gaa)	1,1*	1	1	1	1
*trnG*(gcc)	1,1*	1	1	1	1
*trnG*(ucc)	1,1*	2	1	1	1
*trnH*(gug)	1,1*	1	1	1	1
*trnI*(aau)	1*				
*trnI*(cau)	1	1	1	1	
*trnI*(gau)	1	1	1	2	2
*trnK*(cuu)	1*				
*trnK*(uuu)	1	1	1	1	1
*trnL*(caa)	1*		1	1	
*trnL*(uaa)	1	1	1	1	1
*trnL*(uag)	1	1	1	1	1
*trnL*(gag)			1		
*trn*M(cau)^a^	1*				
*trnMe*(cau)	1	1	1	1	1
*trnMf*(cau)	1	1	1	1	1
*trnN*(guu)	1,1*	1	1	1	1
*trnP*(agg)	1*				
*trnP*(ugg)	1,1*	1	1	1	1
*trnQ*(cug)	1*				
*trnQ*(uug)	1	1	1	1	1
*trnR*(acg)	1,1*	1	1	1	1
*trnR*(ucg)	1*				
*trnR*(ccu)	1*			1	1
*trnR*(ucu)	1	1	1	1	1
*trnS*(gcu)	1,1*	1	1	1	1
*trnS*(uga)	1	1	1	1	1
*trnT*(agu)	1*				
*trnT*(ugu)	1	1	1	1	1
*trnV*(acc)	1*				
*trnV*(cac)	1*				
*trnV*(uac)	1	1	1	1	1
*trnW*(cca)	1,1*	1	1	1	1
*trnY*(gua)	1,1*	1	1	1	1
**Total**	**53**	**27**	**28**	**27**	**27**
**Unique**	**40**	**26**	**28**	**27**	**27**

^a^The *trnM*(cau) gene situated in the large tRNA region in *B. hypnoides* showed similarity (blastn E value < 2e-10) with land plant nuclear encoded tRNA-Met initiator (*Met-tRNA-i*) genes.

Numbers indicate gene copy. An asterisks indicates that the gene or gene copy is situated in the large tRNA region in *B. hypnoides* (Additional file 2). *B. hyp* = *Bryopsis hypnoides*, *B. plu* = *B. plumosa*, *T. exp* = *Tydemania expeditiones*, *P. aki* = *Pseudendoclonium akinetum*, *O. vir* = *Oltmannsiellopsis viridis*.

**Table 3:** **Freestanding ORFs in** ***Bryopsis plumosa*** **and** ***Tydemania expeditiones*** **cpDNAs**

**Species orf number**	**Length (AA)**	**Closest BLAST hit(s)**	**blastp E-value**	**Note**
*B. plumosa orf3*	2,676	bacterial *rhs*-family proteins	<1e-39	No similar green algal sequences^a^ (apart from *B. hypnoides*)
*B. plumosa orf4*	212	hypothetical protein, *Francisella* spp.	1e-26	No similar green algal sequences^a^
*B. plumosa orf5*	76	hypothetical protein, *Enterobacter* sp.	4e-19	No similar green algal sequences^a^ (apart from *B. hypnoides*)
*B. plumosa orf7*	446	Bacterial RNA-directed DNA polymerase	<1e-32	Contains a group II intron, maturase-specific domain. Similarity to a RT group II intron protein of *Caulerpa filiformis* (also present in *B. hypnoides*).
*T. expeditiones orf3*	375	Bacterial RNA-directed DNA polymerase	<1e-23	Contains a Group II intron, maturase-specific domain. Similarity with RT in *Caulerpa filiformis* cpDNA
*T. expeditiones orf8*	96	bacterial DNA methyltransferase	<1e-22	Contains Cytosine-C5 specific DNA methylase domain. No similar green algal sequences^a^
*T. expeditiones orf9*	202	bacterial DNA methyltransferase	<1e-56	Contains Cytosine-C5 specific DNA methylase domain. No similar green algal sequences^a^
*T. expeditiones orf13*	287	bacterial transposase	<1e-23	Contains an integrase core domain protein. No similar green algal sequences^a^
*T. expeditiones orf14*, *orf15*	117, 177	bacterial DNA polymerase	< 1e-46	Contains a DNA polymerase family A domain. No similar green algal sequences^a^
*T. expeditiones orf16*	381	bacterial DNA primase or phage/plasmid primase	< 1e-97	No similar green algal sequences^a^

^a^To verify whether homologous genes were present in green algae, blastp searcher were performed with organisms constrained to Viridiplantae (taxid:33090).

Most genes found in *B. plumosa*, with the exception of *ycf20* and *orf4*, were also found in *B. hypnoides* [13] (Additional file 2). However, the cpDNA of *B. hypnoides* contains a region of about 6 kb including 27 tRNA genes, ten of which have not been found in any other green algal cpDNA [13]. This large tRNA region is not present in *B. plumosa*, nor is the set of unique tRNA genes (Table 2). As in *B. plumosa*, several ORFs in *B. hypnoides* showed high similarity to bacterial genes, including a putative *rhs* gene, and genes containing domains commonly found in mobile elements, such as reverse transcriptase, endonuclease and maturase (Additional file 2). Reverse transcriptase proteins catalyze the reverse transcription of RNA to complementary DNA, and have a key role in the mobility of retroviruses, retrotransposons, and organellar group II introns [27,28]. Endonuclease-like proteins catalyze cleavage of retrotransposons before transcription [29], and are also involved in mobility and homing of introns [30]. Maturase-like proteins are best known from bacterial and organellar introns, where they aid in intron splicing [30].

The *T. expeditiones* cpDNA contains 125 unique genes, including 94 protein coding genes, three rRNA genes, and 28 tRNA genes (Tables 1, 2, Figure 4). In addition to the conserved set of chloroplast genes, several freestanding ORFs were found coding for bacterial or intron related genes (Table 3). *Orf3* contains a maturase-specific domain, which are usually found within intronic ORFs. Two overlapping ORFs (*orf8* and *orf9*) are related to bacterial C-5 cytosine-specific DNA methyltransferase genes, a family of genes coding for enzymes that catalyzes the methyl-transfer reaction in the process of DNA methylation, and in bacteria are often associated with restriction/modification systems, which function as a defense against infection of bacteria by bacteriophages [31]. DNA methyltransferases have up till now not been found in plastid genomes. However, the identification of DNA methyltransferase genes in organelles is not unprecedented as a gene of this nature has been identified in the mitochondrial genome of the streptophyte *Klebsormidium*, which was seen as a possible remnant of viral infection [32]. A region of about 7 kp contains four freestanding ORFs showing close similarity to bacterial genes involved in mobile functions. *Orf13* showed high similarity to bacterial transposase and contains an integrase core domain. Transposases and integrases catalyze the movement and integration of DNA copies to new locations within and between genomes [33]. *Orf14* and *orf15* showed similarity to bacterial DNA polymerase, containing a DNA polymerase family A domain. Family A polymerases fill DNA gaps that arise during DNA repair, recombination and replication, and are found primarily in prokaryotes [34]. *Orf16* showed high similarity to phage/plasmid DNA primases, which are known from phages and plasmids of Bacteria and Archaea [35]. The presence of these four ORFs indicates that the 7 kp region may be a mobile element of bacterial origin.

The presence of bacterial genes (including genes involved in mobile functions) in both the *Bryopsis* and *Tydemania* cpDNAs, and absent in other green algae, suggest that these genes have been acquired through horizontal gene transfer. Transfer of genes to plastid genomes is only rarely observed but some clear instances are known [36,37]. In the *Oedogonium* chloroplast genome, the unprecedented finding of *int* (a gene belonging to the family of tyrosine recombinases) and *dpoB* (a member of the B family of DNA-directed DNA polymerases) was seen as evidence of horizontal transfer, possibly from a mitochondrial genome donor [99]. In the cpDNA of *Nephroselmis olivacea*, two large regions (27 kb in total) are believed to have been acquired by lateral transfer from a bacterial donor based on a deviant base composition, the lack of genes typically found in cpDNAs, and the presence of an ORF showing similarity to phage associated DNA primases [9,38]. Horizontal gene transfer from bacteria to plastids has also been demonstrated in other groups of algae, including red algae [39], dinoflagellates [37,40], haptophytes and cryptophytes [41,42]. It is relevant to note that siphonous green algae (Bryopsidales) are known to harbor intracellular bacterial communities, with some bacteria showing close associations with the host (e.g., [43-45]). Based on fluorescence in situ hybridization, bacteria in *Bryopsis* were found in the vacuole or in the cytoplasmic layer of the siphonous cells, sometimes closely adhered to the chloroplast membrane [46]. These intracellular bacteria may facilitate gene transfer from the endobiontic bacteria to the host genome, possibly via vectors such as bacteriophages.

The two *Bryopsis* cpDNAs share nearly identical gene repertoires with *T. expeditiones*, except for *rpl12*, which is present in *B. plumosa* and *B. hypnoides* but was not found in *T. expeditiones*. In addition, several of the ORFs discussed above were uniquely found in *B. plumosa* or *T. expeditiones* (Table 3). A number of genes in *Bryopsis* and/or *Tydemania*, including *psbM*, *rpl19*, and *rpl23*, were found to be quite divergent from core chlorophytan orthologs based on visual inspection of amino acid alignments and branch lengths of resulting phylogenetic trees (Additional file 3). The *petL* gene is present in two identical copies in *B. plumosa* (see inverted repeats below). Also *B. hypnoides* contains two copies of *petL*, but here they differ in nucleotide composition as well as in length: a 96 bp copy identical to that of *B. plumosa*, and a 105 bp copy that is divergent from the bryopsidalean orthologs (Additional file 4). *Tydemania expeditiones* contains a single 96 bp copy of *petL*.

A comparison of gene repertoires between *B. plumosa*, *T. expeditiones* and 18 published core chlorophytan cpDNAs is shown in Figure 4. A total of 80 genes are shared among the 20 cpDNAs. A large proportion of genes are shared among species of Ulvophyceae and Trebouxiophyceae, while most Chlorophyceae have a smaller gene repertoire (Figure 4). Notably, several genes are shared between the Bryopsidales and Trebouxiophyceae, but are absent from *Pseudendoclonium* and/or *Oltmannsiellopsis*. These include three genes encoding subunits of protochlorophyllide reductase (*chlB*, *L*, *N*), tRNA Ile-lysidine synthetase (*tilS*) and hypothetical protein *ycf47*. Only one gene, the organelle division inhibitor factor, *minD*, was present in *Pseudendoclonium*, *Oltmannsiellopsis*, and Trebouxiophyceae but absent in Bryopsidales.

tRNA(Ile)-lysidine synthase (*tilS*), also known as *ycf62*, is present in all Ulvophyceae and Trebouxiophyceae, but absent from Chlorophyceae (Figure 4). In *T. expeditiones*, this gene seems to be pseudogenized by a stop codon at amino acid position 152 (492 bp) from the start methionine. A similar situation was found in *Caulerpa filiformis* (like *Tydemania* a member of the Halimedineae), which contains a *til*S pseudogene with a frame shift at about the same position [16]. In *B. plumosa* and *B. hypnoides* the gene is interrupted by a 71 bp, AT-rich (80.3 and 81.7% AT) insertion at amino acid position 157.

### Introns

In *B. plumosa* 13 introns are present in 12 genes, comprising 8.3% (including intronic ORFs) of the total cpDNA (Tables 1 and 4). Four introns were identified as group I introns, and five as group II introns, while for four introns the class could not be determined with certainty. In *T. expeditiones* 11 introns are present in 7 genes, comprising 11.2% (including intronic ORFs) of the total cpDNA. Seven introns were identified as group I introns, and two as group II introns, while the class of two introns could not be determined with certainty. Several of the introns in *B. plumosa* and *T. expeditiones* (*psaA*, *psbB*, *rrl* and *rrs* introns) are commonly found in orthologous genes of other green algae. Others are only rarely found in green algae or have not yet been observed in plastids in general. These are discussed below.

**Table 4:** **Distribution and characteristics of introns in** ***Bryopsis plumosa*** **and** ***Tydemania expeditiones***

**Gene**	***B. plu***	***T. exp***	**Length (** ***B. plu*** **/** ***T. exp*** **) (bp)**	**Insertion positions** ^**a**^	**AT content (excl. intronic ORF) (** ***B.plu*** **/** ***T.exp*** **)**	**Intronic ORF**	**Class**
*atpA* (intron 1)	+	-	239	489	69%	-	group I intron
*atpA* (intron 2)	+	-	728	753	70%	-	putative group II
*atpF*	+	+	481/373	108/108	82/77%	-	undetermined
*ccsA*	+	+	385/370	210/210	79/78%	-	undetermined
*psaA*	+	-	2244	1797	69%	RT, IM	group II
*psbA*	-	+	3227	576	67%	RT, IM	group II
*psbB*	+	-	1103	600	73%	LHE	group I
*psbC*	-	+	2491	1131	57%	RT, IM	group II
*psbT*	+	-	427	27	82%	-	undetermined
*rpl5*	+	-	389	171	77%	-	putative group II
*rpl23*	+	-	351	24	85%	-	undetermined
*rps19*	+	-	848	141	64%	-	putative group II
*rrl* (intron 1)	-	+	776	1917	73%	LHE	group I
*rrl* (intron 2)	-	+	801	1923	69%	LHE	group I
*rrl* (intron 3)	-	+	1049	1931	67%	LHE ^b^	group I
*rrl* (intron 4)	+	+	1037/730	2598/2598	63/71%	LHE	group I
*rrs* (intron 1)	-	+	860	510	67%	LHE	group I
*rrs* (intron 2)	-	+	988	794	71%	LHE	group I
*trnL*(uaa)	+	+	205/152	35	73/65%	-	putative group I
*ycf3*	+	-	373	174	79%	-	putative group II

^a^Intron insertion site positions correspond to the nucleotide immediately preceding the intron. Insertion sites in genes coding for proteins and the tRNA are given relative to the corresponding genes in *Mesostigma viride* cpDNA (GenBank NC_002186); insertion sites in the rRNA genes are given relative to the 16S and 23S rRNA genes of *Escherichia coli* (GenBank NC_004431).

^b^Contains two LAGLIDADG homing endonuclease domains.

RT = reverse transcriptase, IM = intron maturase, LHE = LAGLIDADG homing endonuclease.

The *atpA* gene in *B. plumosa* contains two introns. The first was identified as a group I intron, which is commonly found in this gene in other green algae [4,12]. The second intron was identified as a putative group II intron (Additional file 5), which has until now only been found in the *atpA* gene of *Volvox carteri*.

The *psbA* gene in *T. expeditiones* has a large intron containing two overlapping ORFs: one encoding reverse transcriptase and intron maturase, the other encoding N-terminal domain of reverse transcriptase. The majority of *psbA* introns in green algae are group I introns. Thus far, only one other green algal *psbA* group II intron has been identified, in *Floydiella terrestris* [7]. The *psbC* intron in *T. expeditiones* was also identified as a group II intron based on the presence of an ORF encoding reverse transcriptase and intron maturase. Introns are common in green algal *psbC* genes, but are all group I introns. *PsbC* group II introns have thus far only been found in euglenoids [47,48].

The *rpl5* and *rps19* introns in *B. plumosa* are putative group II introns (RNA secondary structure showed 6 helices, helix V with conserved AGC, and helix VI with bulged A). Thus far, *rpl5* introns have only been found in *B. hypnoides*. Plastid *rps19* introns were until now only known in euglenoids (where they have been identified as group III introns) [48,49] and the red alga *Porphyridium purpureum* (unspecified intron class) [50]. These introns ranged between 95-216 bp in size, which is much smaller than the intron found in *B. plumosa*.

AT-rich introns (75-85% AT) of which the class could not be determined with certainty were found in several genes, including *atpF*, *ccsA*, *psbT* and *rpl23. AtpF* introns are also present in some charophytic green algae, where they have been identified as group II introns [23,51]. Introns in plastid *rpl23* were until now only known in euglenoids (group III intron) and *B. hypnoides*. The length of the *Bryopsis rpl23* intron falls within the range of those found in euglenoids (47-440 bp), and the high AT-content is similar to the euglenoid introns (76-91% AT). *B. plumosa* contains an AT-rich intron in the *psbT* gene. Introns in this gene have thus far only been found in euglenoids (all group II). Another AT-rich intron was found in the *ccsA* gene of *B. plumosa* and *T. expeditiones*, which is unexpected as this gene lacks introns in all other published plastids. Similar to the other AT-rich introns, the class could not be determined because we failed to identify conserved structural motives.

The *trnL*(uaa) gene in *Bryopsis* and *Tydemania* contains a group I intron. This intron has been assumed to have been present in the common ancestor of Cyanobacteria and chloroplasts because it is found across a wide diversity of Cyanobacteria and plastids where it is conserved in position, secondary structure and primary sequence [52]. In green algae, this intron is present in several charophytes, and core Chlorophyta, but is absent in the published cpDNAs of prasinophytes [3] and the ulvophytes *Pseudendoclonium* and *Oltmannsiellopsis* [11,12]. The absence from the early diverging prasinophytes implies multiple losses of the *trnL* intron.

### Repeats

**Figure 5:**
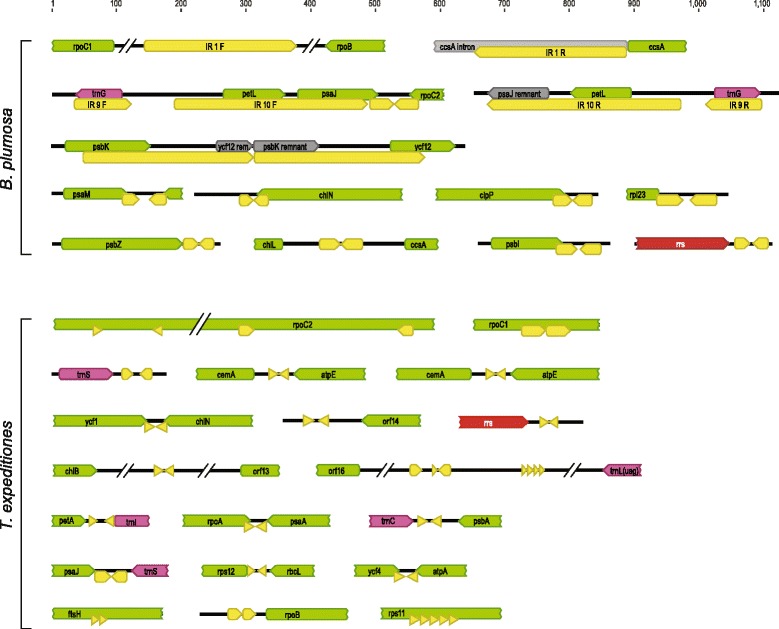
**Sequence repeats (yellow) in** ***Bryopsis plumosa*** **and** ***Tydemania expeditiones*** **cpDNAs.** Arrows indicate the direction of the repeats. Protein coding genes are indicated in green, tRNA genes in purple and rRNA genes in red.

The *B. plumosa* cpDNA contains three relatively large inverted repeats (IRs) and one large tandem repeat (Figure 5). IR1 (237 bp) duplicates part of the *ccsA* intron in an intergenic region between *rpo*C1 and *rpoB*. IR9 (88 bp) duplicates *trnG*(ucc), and IR10 (300 bp) duplicates *petL* and part of *psaJ*. A tandem repeat of 2 × 265 bp duplicates part of *psbK* and part of *ycf12*.

In *T. expeditiones* several repeats are present within CDSs: *rpoC2* contains two inverted repeats of 15 and 25 bp long, *rpoC1* contains a tandem repeat of 2 × 38 bp, and *rps11* contains a tandem repeat of 5 × 15 bp (Figure 5). The intergenic spacer region between *orf16* and *trnL*(uag) contains a short inverted repeat and a tandem repeat of 5 × 9 bp.

In addition, *B. plumosa* and *T. expeditiones* contain numerous short inverted repeats near the 3’end of genes or overlapping with the 3’end of genes (Figure 5). Short IRs in the 3’untranslated regions (UTRs) of mRNAs are common in organellar genomes where they serve as RNA-processing signals [53-55]. In *B. plumosa* and *T. expeditiones*, several of these short IRs partially overlap with the 3’-end of the CDSs (e.g. *psaM*, *chlN*, *clpP*, *rpl23*, *psbI* in *B. plumosa*, and *ycf1*, *chlN*, *rpoA* in *T. expeditiones*), a condition also found in bacteria where hairpins preceding the stop codon are involved in termination of transcription by RNA polymerase at rho-independent sites, known as intrinsic termination [56].

### Synteny

**Figure 6:**
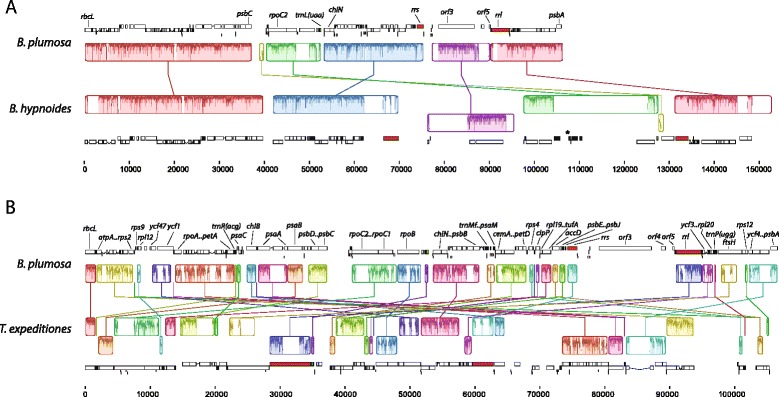
**Alignments between the chloroplast genomes of** ***Bryopsis plumosa*** **,** ***B. hypnoides*** **, and** ***Tydemania expeditiones*** **.** The Mauve algorithm [81] was used to align the cpDNAs between *B. plumosa* and *B. hypnoides* **(A)**, and between *B. plumosa* and *T. expeditiones* **(B)**. Corresponding colored boxes are locally collinear blocks (LCBs), which represent homologous regions of sequences that do not contain any major rearrangements. Inside each block a sequence identity similarity profile is shown. Inverted LCBs are presented as blocks below the center line. Annotations are shown above and below the LCBs: protein coding genes as white boxes, tRNA genes in green, rRNA genes in red and short repeats in pink. Lowered position of a box indicates inverted orientation. The asterisk indicates the large tRNA region in *B. hypnoides*.

The Mauve alignments between the two *Bryopsis* cpDNAs and between *B. plumosa* and *T. expeditiones* (Figure 6) visualize locally collinear blocks (LCBs), representing homologous regions of sequences that do not contain any major rearrangements. The syntenic structure of the *B. plumosa* cpDNA is similar to that of *B. hypnoides*, separated by 25 rearrangements as calculated by the double cut and join (DCJ) analysis. When excluding all tRNA genes from the analysis, the DCJ distance is reduced to 7. This large difference is mainly due to numerous inversions of tRNA genes, and the absence of the large tRNA region in *B. plumosa*. The cpDNAs of the two *Bryopsis* species and *Tydemania* are more rearranged with respect to each other, with a DCJ distance of 36 (23 excluding all tRNA genes). This high dissimilarity in synteny is not surprising as high variability in cpDNA architecture has also been observed among species of Chlorophyceae and Trebouxiophyceae, including between congeneric taxa [4,22,57]. Even higher number of rearrangements separate the cpDNAs of Bryopsidales and *Pseudendoclonium* and *Oltmannsiellopsis* (DCJ distances up to 60).

**Figure 7:**
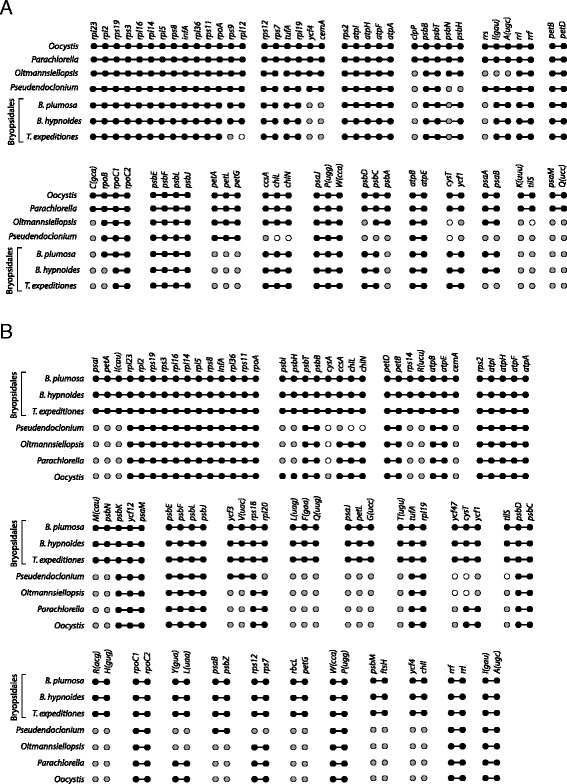
**Comparison of conserved gene clusters. (A)** Comparison of gene clusters conserved between at least two of the four depicted species, representing different ulvophycean and trebouxiophycean lineages [based on Turmel et al. [22]] with gene order found in the Bryopsidales (*Bryopsis plumosa, B. hypnoides* and *Tydemania expeditiones*). **(B)** Comparison of gene clusters conserved in Bryopsidales with gene order found in four species, representing different ulvophycean and trebouxiophycean lineages. Black connected circles indicate gene clusters. Grey circles indicate genes that are located elsewhere on the cpDNA. White circles indicated genes that are missing from the cpDNA.

Several, but not all, gene clusters conserved in the Ulvophyceae and Trebouxiophyceae [based on Turmel et al. [22]] are also conserved in *Bryopsis* and *Tydemania*. One notable exception is the *petA*-*petL*-*petG* cluster, which is absent in the Bryopsidales (Figure 7A). On the other hand, some gene clusters shared in the Bryopsidales are not present in any of the four analyzed Ulvophyceae and Trebouxiophyceae species (Figure 7B).

### Phylogenomic analyses

**Figure 8:**
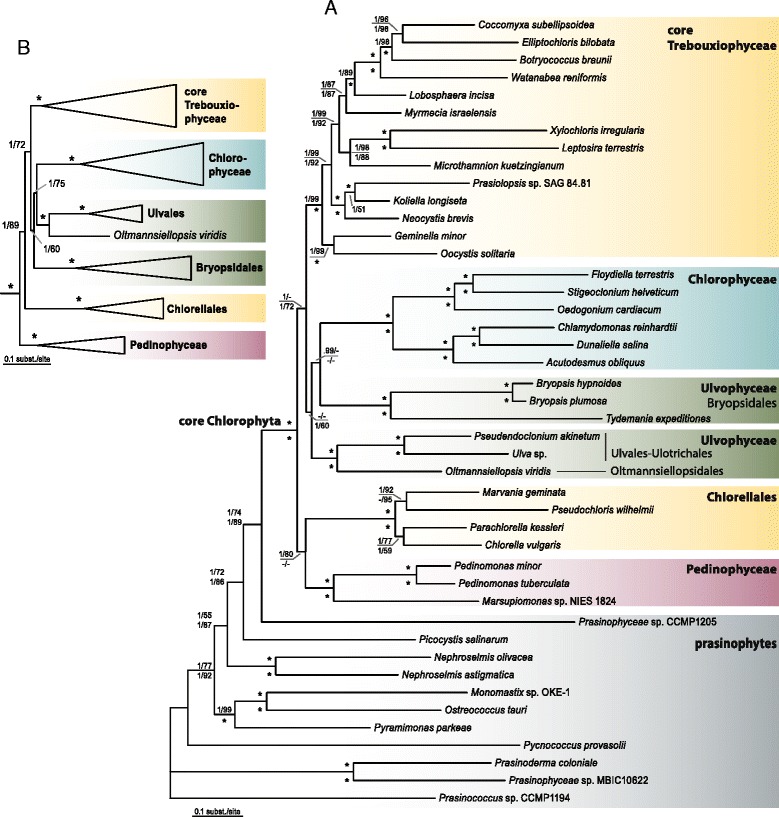
**Phylogenetic trees of Chlorophyta inferred from a 79-gene dataset. (A)** Bayesian majority rule tree showing all compatible partitions, inference from the protein alignment of 79 concatenated chloroplast genes (16,205 amino acid positions and 44 terminal taxa). Node support is given as Bayesian posterior probabilities and maximum-likelihood (ML) bootstrap values of the protein analyses (above branches), and the nucleotide analyses (below branches); values <0.9 and 50, respectively, are not shown; asterisks indicated full support in both the Bayesian and ML analyses. **(B)** Bayesian tree inference from the nucleotide alignment (first two codon positions) of 79 concatenated chloroplast genes (32,410 nucleotide positions and 44 terminal taxa). Node support is given as Bayesian posterior probabilities and maximum-likelihood (ML) bootstrap values, and asterisks indicate full support in both analyses.

The phylogenetic trees resulting from the analyses of the 79-gene dataset are summarized in Figure 8. The topology of the trees are in general agreement with published chloroplast phylogenies of Chlorophyta [3,18,19]. Among prasinophytes, the position of *Pycnococcus provasolii* differs from the analyses of Lemieux et al. [3,19], but this may be attributable to a difference in tree rooting. The core Chlorophyta are comprised of several well supported main clades, including the Pedinophyceae, Chlorellales, the core Trebouxiophyceae, Chlorophyceae and two clades of Ulvophyceae. The relationships among these lineages are generally poorly supported, which contrasts with previous analyses that did not include members of the Bryopsidales [19]. Although the phylogenies recovered two separate Ulvophyceae clades, the relationship between the two is poorly supported and therefore our analyses are inconclusive with respect to monophyly of the class. The unstable phylogenetic position of the Bryopsidales is also apparent when comparing published chloroplast multi-gene phylogenies, which revealed different relationships depending on gene and taxon sampling: a sister relationship between *Bryopsis* and Chlorophyceae [13], between *Caulerpa* (Bryopsidales) and *Chlorella* [16], or between the core Trebouxiophyceae and a Bryopsidales + Dasycladales + Trentepohliales clade [18].

**Figure 9:**
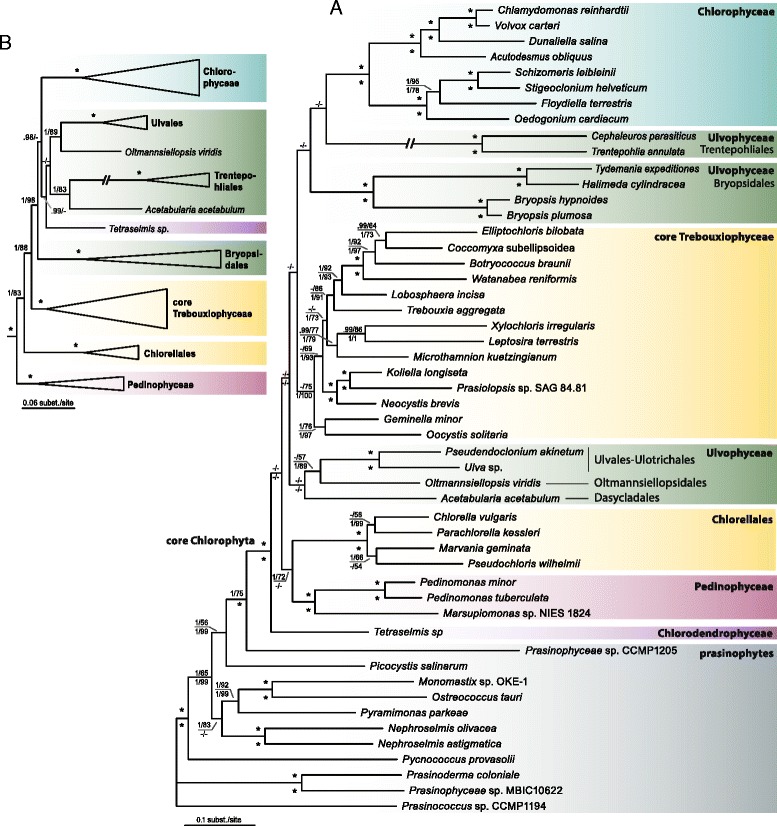
**Phylogenetic trees of Chlorophyta inferred from a 50-gene dataset. (A)** Bayesian majority rule tree showing all compatible partitions, inference from the protein alignment of 50 concatenated chloroplast genes (9,300 amino acid positions and 51 terminal taxa). Node support is given as Bayesian posterior probabilities and maximum-likelihood (ML) bootstrap values of the protein analyses (above branches), and the nucleotide analyses (below branches); values <0.9 and 50, respectively, are not shown; asterisks indicated full support in both the Bayesian and ML analyses. **(B)** Bayesian tree inference from the nucleotide alignment (first two codon positions) of 50 concatenated chloroplast genes (18,600 nucleotide positions and 51 terminal taxa). Node support is given as Bayesian posterior probabilities and maximum-likelihood (ML) bootstrap values, and asterisks indicate full support in both analyses.

The phylogenetic trees resulting from the analyses of the 50-gene dataset are summarized in Figure 9. The 50-gene dataset differs from the 79-dataset mainly in the inclusion of species of Trentepohliales and Dasycladales (both orders of Ulvophyceae) and *Tetraselmis* (Chlorodendrophyceae). Overall, the 50-gene phylogenies recovered the same main clades as the 79-gene phylogenies, in addition to two extra clades of Ulvophyceae (Trentepohliales and Dasycladales), and *Tetraselmis*. Similar to the 79-gene phylogenies, the relationships among the main clades of core Chlorophyta (including the different clades of Ulvophyceae) received little statistical support, and therefore the monophyly of the Ulvophyceae cannot be confirmed nor rejected by our data. The phylogenies inferred from the nucleotide alignment (Figure 9B) recovered the same main clades as the protein analyses, but the topology and branch support differs in several aspects, including a well-supported Trentepohliales + Dasycladales clade, and a different phylogenetic position of *Tetraselmis*.

The relationships within the Ulvophyceae and the monophyly of the class has been the subject of a longstanding debate [2,17,18,21]. Phylogenies based on nuclear ribosomal DNA data have remained ambiguous and often recovered two distinct clades: the Oltmannsiellopsidales + Ulvales-Ulotrichales clade, and a clade consisting of Trentepohliales, Cladophorales, Bryopsidales and Dasycladales (TCBD clade) [21,58-61]. Similarly, multigene chloroplast data have failed to provide support for monophyly of the Ulvophyceae [13,16,18]. Conversely, phylogenetic analyses based on 10 genes (eight nuclear- and two plastid-encoded) recovered the class as a monophyletic group with strong support [17,61]. These analyses also supported the division of the two above mentioned distinct ulvophycean clades. Within the TCBD clade, the Bryopsidales and Dasycladales were recovered as sister lineages with high support. The division of two distinct ulvophycean clades was also supported by independent molecular data, such as the distribution of elongation factor genes [60,62], and a non-canonical nuclear genetic code [63]. It is important to note that several important clades that were present in the analyses of Cocquyt et al. [17] and Škaloud et al. [61], such as the Cladophorales, Scotinosphaerales, *Blastophysa*, and *Ignatius*-clade, were not included in our study, which may have an important effect on phylogenetic reconstruction. A notable difference between our phylogenies and the ones of Fučíková et al. [18] is the position of *Oltmannsiellopsis* and *Tetraselmis. Oltmannsiellopsis* is currently classified as an ulvophyte based on morphological and molecular data [59,60,64]. *Tetraselmis* is a member of the Chlorodendrophyceae and has, in nuclear ribosomal-based phylogenies, been recovered as an early branching clade of the core Chlorophyta [2,65,66]. In contrast, chloroplast multi-gene analyses have recovered a clade uniting *Tetraselmis* and *Oltmannsiellopsis*, which was sister to the Ulvales-Ulotrichales [67] or branched early in the radiation of the core Chlorophyta [18]. Our analyses recovered *Oltmannsiellopsis* as sister to the Ulvales-Ulotrichales (Figures 8 and 9), similar to [13,16,19], while the position of *Tetraselmis* remained ambiguous, branching early in the core Chlorophyta in the protein tree (Figure 9A), or branching in the vicinity of ulvophycean lineages in the nucleotide-based phylogeny (Figure 9B). It should be noted that gene sampling in *Tetraselmis* was low, which may explain its unstable phylogenetic position.

An accurate phylogenetic reconstruction of the core Chlorophyta, and in particular the Ulvophyceae will require a richer sampling of taxa and genes. As has been shown in the Trebouxiophytes, expanding taxon sampling has greatly aided in resolving phylogenetic relationships [18,19]. Compared with other groups of core Chlorophyta, the Ulvophyceae are still underrepresented in chloroplast phylogenomic studies (Figure 1), and for several important clades chloroplast genomic data is lacking altogether (e.g. Scotinosphaerales, Cladophorales, *Blastophysa*, and *Ignatius*-clade). Although chloroplast genomes contain large amounts of genetic information, a combination of plastid and nuclear genomic data may be needed to test monophyly of the Ulvophyceae, resolve relationships within the Ulvophyceae, and among the main clades of core Chlorophyta.

## Conclusions

The gene dense chloroplast genomes of *Bryopsis plumosa* and *Tydemania expeditiones* are amongst the smallest chloroplast genomes in the core Chlorophyta. Comparison of the cpDNA of *Bryopsis plumosa* with the previously published cpDNAs of Ulvophyceae highlighted some exceptional differences in genome size, gene content, and gene order, illustrating the remarkable plasticity of green algal chloroplast genomes, even between congeneric species.

The cpDNAs of *B. plumosa*, *B. hypnoides* and *T. expeditiones* contain DNA regions that contain open reading frames showing no affinity to conserved plastid genes, but instead having significant similarity to bacterial genes. These genes include *rhs*-family genes, and several genes involved in mobile functions, such as transposases, integrases, DNA polymerases, and phage/plasmid DNA primases. Another unexpected finding was the presence of two ORFs in *T. expeditiones* showing close similarity to bacterial DNA methyltransferases, a family of genes that have up till now not been found in plastid genomes. The presence of DNA regions including genes with clear bacterial affinity suggests that these regions may have been acquired through horizontal DNA transfer from bacterial donors. Siphonous green algae are characterized by giant tubular cells that contain millions of nuclei and plastids, in addition to diverse bacterial communities. It is imaginable that these horizontal DNA transfers have been facilitated by the occurrence of bacteria residing inside the host cell. Our data adds to the scarce knowledge of horizontal transfer of bacterial DNA to plastid genomes.

## Methods

### Collections

Plant material of *Bryopsis plumosa* was obtained from a culture isolated by John West from Traon Erch, Brittany, France on 5 April 2008 (culture number 4718, now maintained in the algal culture collection of the Phycology Research Group, Ghent University). The specimen was morphologically identified as *B. plumosa*. Molecular data, however, has indicated that many morphospecies in the genus *Bryopsis* (including *B. plumosa*) are polyphyletic. Based on analysis of *rbcL* sequences, the culture 4718 is a member of species clade number 4 in Hollants et al. [68]. For convenience, however, we will use the name *B. plumosa* throughout the paper. Plant material of *Tydemania expeditiones* was obtained from a silica dried specimen collected by FL from Siquijor, Philippines on 17 September 2007 (voucher specimen FL1151, deposited in the algal herbarium of Ghent University, GENT).

### Genome sequencing and assembly

Total genomic DNA was extracted by using a modified CTAB extraction protocol based on Doyle & Doyle [18,69]. Sequencing of the genomic DNA was performed by Cold Spring Harbor Laboratory (Cold Spring Harbor, NY, USA) using Illumina HiSeq 2000 technology on 1/5^th^ of a lane. The sequencing run generated 7.2 million paired-end reads (2 × 101 bp) for *B. plumosa* and 14.7 million paired-end reads for *T. expeditiones*.

Low-quality ends of the reads (Phred score < 30) and adapters were trimmed using Trim Galore! (www.bioinformatics.babraham.ac.uk/projects/trim_galore), and only reads longer than 2 × 94 bp were retained. De novo assembly was performed from the paired-end Illumina reads using three different assembly programs: (1) Velvet v. 1.2.10 [70] using a kmer = 93, (2) Geneious v. 7 (Biomatters, www.geneious.com) de novo assembler using “medium sensitivity”, and (3) CLC Genomics workbench v. 6 (www.clcbio.com) de novo assembler using word size = 63 and default parameters.

Contigs corresponding to cpDNA sequences were extracted from the total assembly using blastn similarity searches (E-value < 10^-6^) of a custom build blast database including all genes from published cpDNAs of Chlorophyta and *Mesostigma*. The three assemblies (Velvet, Geneious and CLC) yielded similar results (i.e. cpDNA contigs with identical sequences, but different in length). Scaffolds were constructed by re-assembling the cpDNA contigs using Geneious with stringent conditions (minimum overlap = 80 bp, no gaps allowed, and minimum overlap identity of 95%).

### Gene annotation

Initial annotations were performed by mapping genes from published cpDNAs of Chlorophyta against the two cpDNA contigs in Geneious. This was followed by a more detailed examination of protein-coding genes, rRNA genes, tRNA genes, introns, and additional features such as sequence repeats.

For protein-coding genes, open reading frames (ORFs) were identified by getorf from the EMBOSS suite server [71]. Similarities of the protein sequences were detected using blastp 2.2.30 [72] against all non-redundant GenBank CDS translations + PDB + SwissProt + PIR + PRF (excluding environmental samples from WGS projects). Some ORFs were identified that showed no clear similarity with green algal plastid genes. In order to confirm that homologous genes were absent from green algae, blastp searcher was performed but with organisms constrained to Viridiplantae (taxid:33090).

Intron-exon boundaries were identified by translation alignment of the intron-containing genes with CDSs of orthologous genes from published chlorophytan cpDNAs. In order to identify the intron class (group I or group II), RNA secondary structures of the introns were predicted using Mfold [73], taking into account suboptimal foldings, and compared to the models of Michel et al. [74] and Michel and Westhof [75].

The boundaries of the rRNA genes and possible introns in the rRNA genes were identified based on a data set of complete rRNA genes from published green algal cpDNAs, aligned using MUSCLE [76]. tRNA genes were detected and identified using tRNAscan-SE 1.21 [77].

Repeats and inverted sequence repeats were detected using einverted, etandem and palindrome (EMBOSS suite server), and Tandem repeats finder [78].

We also re-annotated the published cpDNA of *Bryopsis hypnoides* (GenBank accession NC_013359) [13] because our initial comparison with *B. plumosa* and other species of Chlorophyta yielded some unexpected results, such as the lack of some common genes in *B. hypnoides* (e.g. *chlI*, *ftsH*, *psaI*, *psaM*, and *rpl19*).

### Whole-genome alignments and analysis of genome rearrangements

The *Bryopsis* and *Tydemania* cpDNAs were aligned using the progressive Mauve algorithm in Geneious using the full alignment option and automated calculation of locally collinear block (LCB) scores [58,79]. Mauve is a general-purpose genome aligner that identifies LCBs despite small rearrangements and reversals. Minimal histories of rearrangements among the pairwise aligned genomes were predicted using UniMoG [80] under a double cut and join (DCJ) model [81,82], which allows for common rearrangement operations such as inversions, translocations, fusions, and fissions.

### Phylogenomic analyses

Phylogenomic analyses were based on two data sets: 1) a 79-gene alignment of 44 taxa of Chlorophyta, including six Ulvophyceae, and 2) a 50-gene alignment of 51 taxa of Chlorophyta, including ten Ulvophyceae (Additional file 6).

Gene selection for the 79-gene alignment was based on Lemieux et al. [19], and included *accD*, *atpA*, *B*, *E*, *F*, *H*, *I*, *ccsA*, *cemA*, *chlB*, *I*, *L*, *N*, *clpP*, *cysA*, *T*, *ftsH*, *infA*, *minD*, *petA*, *B*, *D*, *G*, *L*, *psaA*, *B*, *C*, *I*, *J*, *M*, *psbA*, *B*, *C*, *D*, *E*, *F*, *H*, *I*, *J*, *K*, *L*, *M*, *N*, *T*, *Z*, *rbcL*, *rpl2*, *5*, *12*, *14*, *16*, *19*, *20*, *23*, *32*, *36*, *rpoA*, *B*, *C1*, *C2*, *rps2*, *3*, *4*, *7*, *8*, *9*, *11*, *12*, *14*, *18*, *19*, *tufA*, *ycf1*, *3*, *4*, *12*, *20*, *47*, *62.* Taxon sampling included 44 taxa of Chlorophyta, based on a selection of taxa from Lemieux et al. [3,19], in addition to *Ulva* sp. UNA00071828 [83], and the two *Bryopsis* species and *Tydemania*. The dataset was 95% filled at the taxon × gene level. The *rpoB*, *rpoC1* and *rpoC2* sequences of *B. hypnoides* were excluded from the dataset because parts of these genes could not be reliably aligned with the chlorophytan orthologs.

Selection of genes for the 50-gene alignment was largely based on Fučíková et al. [18], including *atpA, B, E, F, H, I, infA, petA, B, G, psaA, B, C, J, M, psbA, B, C, D, E, F, I, J, K, L, N, T, rbcL, rpl2, 5, 14, 16, 20, 23, 32, 36, rps3, 4, 7, 8, 9, 11, 12, 14, 18, 19, tufA, ycf3, 4, 12*. The dataset was comprised of 51 taxa of Chlorophyta, including taxa for which complete cpDNAs or partial multigene data were available (Additional file 6). The dataset was 93% filed at the taxon × gene level. Taxa with low gene coverage included *Acetabularia acetabulum* (37 genes), *Cephaleuros parasiticus* (17 genes), *Halimeda cylindracea* (33 genes), *Tetraselmis*_sp. (11 genes), and *Trentepohlia annulata* (18 genes).

DNA sequences were aligned for each gene separately using the ClustalW translational alignment function [84] in Geneious with a BLOSUM cost matrix, and gap open penalty 10 and gap extend cost 0.1. Gene alignments were concatenated, and poorly aligned positions removed using the Gblocks server [85] (http://molevol.cmima.csic.es/castresana/Gblocks_server.html) by preserving the DNA codons, and using the least stringent settings: allowing smaller final blocks, gap positions within the final blocks, less strict flanking positions, and many contiguous non-conserved positions. Gblocks reduced the 79-gene alignment from 140,196 to 48,615 positions, and the 50-gene alignment from 41,166 to 27,900 positions. The resulting alignments were translated to obtain protein alignments of 16,205 amino acid positions (79-gene alignment), and 9,300 amino acid positions (50-gene alignment). For the phylogenetic analyses, only the first two codon positions were included in the nucleotide alignment (32,410 positions for the 79-gene alignment, and 18,600 positions for the 50-gene alignment).

Phylogenetic trees were inferred from the protein and nucleotide alignments using Bayesian and maximum likelihood (ML) analyses. For the protein alignments, a CPREV + Γ4 model was assumed. For the nucleotide alignments, a GTR + Γ4 model was selected, and a partitioning strategy in which codon positions were separated (2 partitions). Bayesian analyses were conducted using MrBayes v.3.2.1 [86], running each analysis for 2 million (protein alignments) or 5 million (nucleotide alignments) generations with one cold and three heated chains, and sampling every 1000 generations. For each analysis, two independent runs were performed. The first 10% of samples was discarded as burn-in based on assessment of convergence of the runs and stability of parameters using Tracer v.1.5 [87]. ML trees were inferred using RAxML v.7.3.5 [88,89] using default parameters. Branch support was assessed by bootstrapping with 500 replicates. All phylogenetic analyses were run on the CIPRES Science Gateway v3.3. [90].

### Availability of supporting data

The two cpDNAs have been deposited in EMBL’s European Nucleotide Archive (ENA) (GenBank/DDBJ) under accession numbers [ENA: LN810504] (*Bryopsis plumosa*) and [ENA: LN810505] (*Tydemania expeditiones*). Phylogenetic data (alignments and phylogenetic trees) have been deposited in TreeBase under accession number 17184 (http://purl.org/phylo/treebase/phylows/study/TB2:S17184).

## Additional files

Additional file 1:
**Read coverage graphs of the cpDNAs of** ***Bryopsis plumosa*** **and** ***Tydemania expeditiones.***
Additional file 2:
**Re-annotated** ***Bryopsis hypnoides*** **cpDNA.**
Additional file 3:
**Amino acid alignment of** ***psbM*** **,** ***rpl19*** **and** ***rpl23*** **and corresponding trees.**
Additional file 4:
***petL*** **amino acid alignment and corresponding phylogenetic tree.**
Additional file 5:
**Putative RNA secondary structure of** ***atp*** **A type II intron of** ***Bryopsis plumosa.***
Additional file 6:
**Taxon sampling for the 79-gene and 50-gene datasets.**

## Abbreviations

CDS: Coding DNA sequence
cpDNA: chloroplast DNA
DCJ: Double cut and join
IR: Inverted repeat
LCB: Locally collinear block
ML: Maximum likelihood
ORF: Open reading frame
