# Supplementary material for: The chloroplast genomes of Bryopsis plumosa and Tydemania expeditiones (Bryopsidales, Chlorophyta): compact genomes and genes of bacterial origin
Source: BMC Genomics. 2015 Mar 17;16(1):204. doi: 10.1186/s12864-015-1418-3 (PMC4487195; doi:10.1186/s12864-015-1418-3)

Additional file 1. Read coverage graphs of the cpDNAs of *Bryopsis plumosa* and *Tydemania expeditiones*.

Read coverage is indicated by the blue area plot; protein-coding genes are indicated in yellow, rRNA genes in red, and tRNA genes in purple.

Bryopsis plumosa

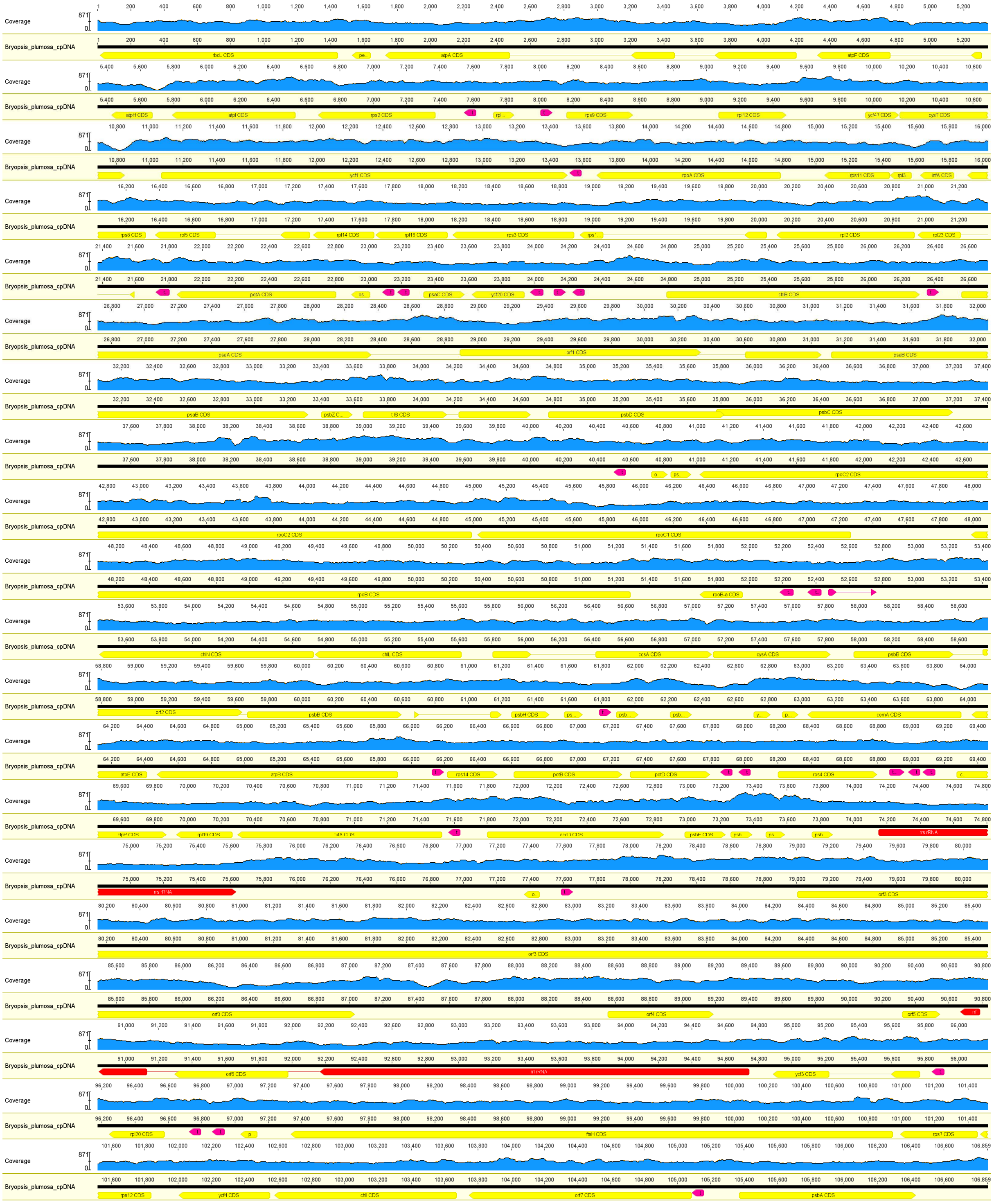

Tydemania expeditiones

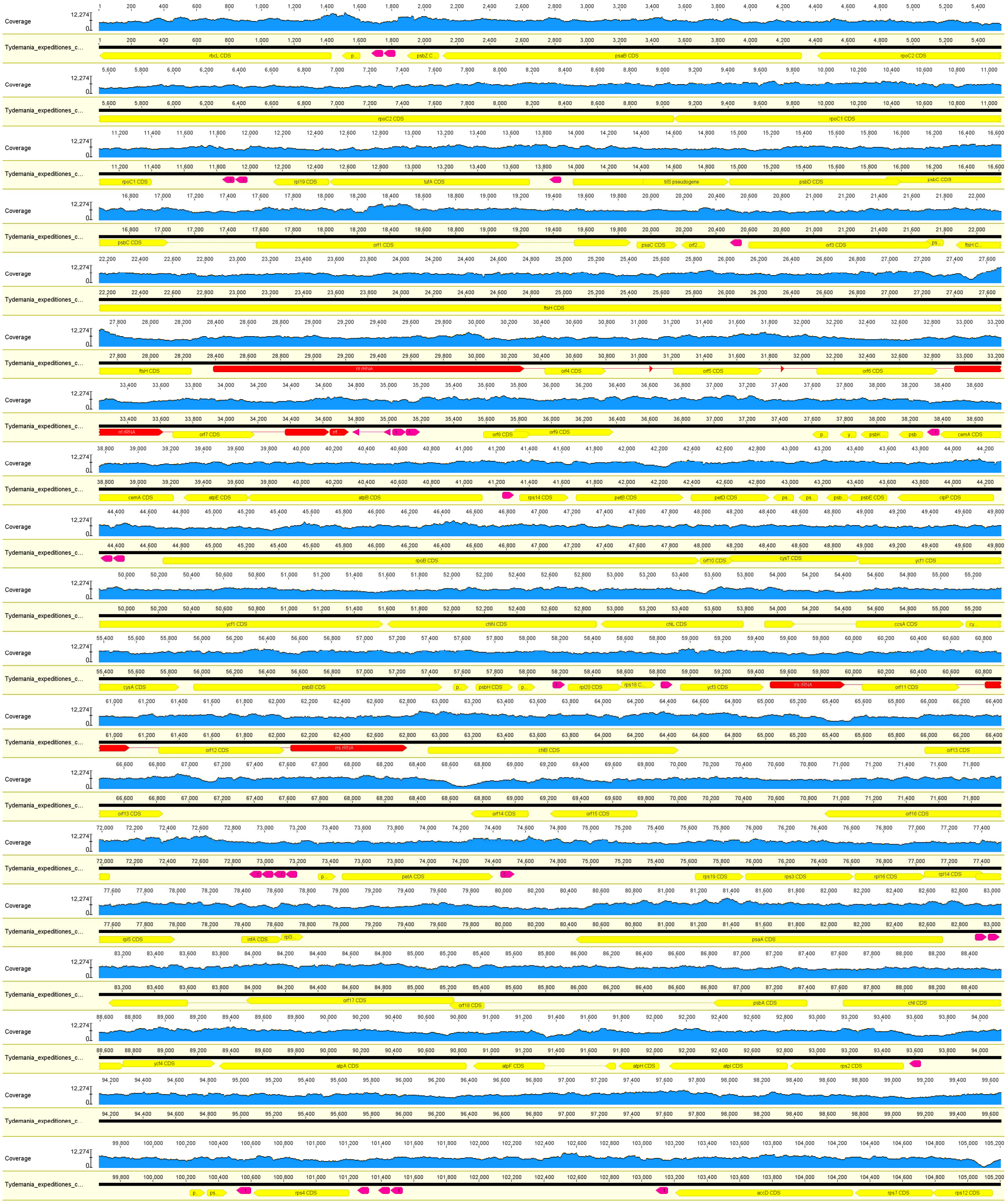

Supplement: Additional file 1: — Read coverage graphs of the cpDNAs of Bryopsis plumosa and Tydemania expeditiones. [file 12864_2015_1418_MOESM1_ESM.pdf]
