# Supplementary material for: The chloroplast genomes of Bryopsis plumosa and Tydemania expeditiones (Bryopsidales, Chlorophyta): compact genomes and genes of bacterial origin
Source: BMC Genomics. 2015 Mar 17;16(1):204. doi: 10.1186/s12864-015-1418-3 (PMC4487195; doi:10.1186/s12864-015-1418-3)

Additional file 2. Re-annotated *Bryopsis hypnoides* cpDNA.

Circular physical map of the chloroplast genome of *Bryopsis hypnoides*. Gene names indicated in red were not included in the original annotation (GenBank accession number NC_013359 = GQ892829) (Lü et al. PLoS One 2011, 6:e14663). To retrieve the *chl*I gene, one position (pos. 11844 in original GenBank file) was deleted to restore the open reading frame (ORF); to retrieve the *rpl*19 genes, one position (pos. 80280) was deleted to restore the ORF; for the *ycf*1 gene, two positions (pos. 36903 and 37229) were deleted to restore the ORF. In the original publication, the rRNA operon was annotated as five coding regions (*rrn*23 = *rrl*, *rrn*16 = *rrs*, *rrn*7 *rrf* = *rrn*5, and *rrn*3). Alignment of green algal *rrl* sequences, however, indicates that the *B. hypnoides rrn*7 and *rrn*3 are part of the *rrl* gene. In addition, the direction of *rrl* was reversed. The re-annotated cpDNA is 153,426 bp long, and contains 131 genes, including 3 ribosomal RNA genes, 39 transfer RNA genes, and 89 protein coding genes. Genes shown on the outside of the circle are transcribed counterclockwise. Annotated genes are colored according to the functional categories shown bottom left. DNA pol. = DNA polymerase, LHE = LAGLIDADG homing endonuclease, mat = maturase, en = endonuclease, Rhs = *Rhs*-family protein gene, rt = reverse transcriptase.

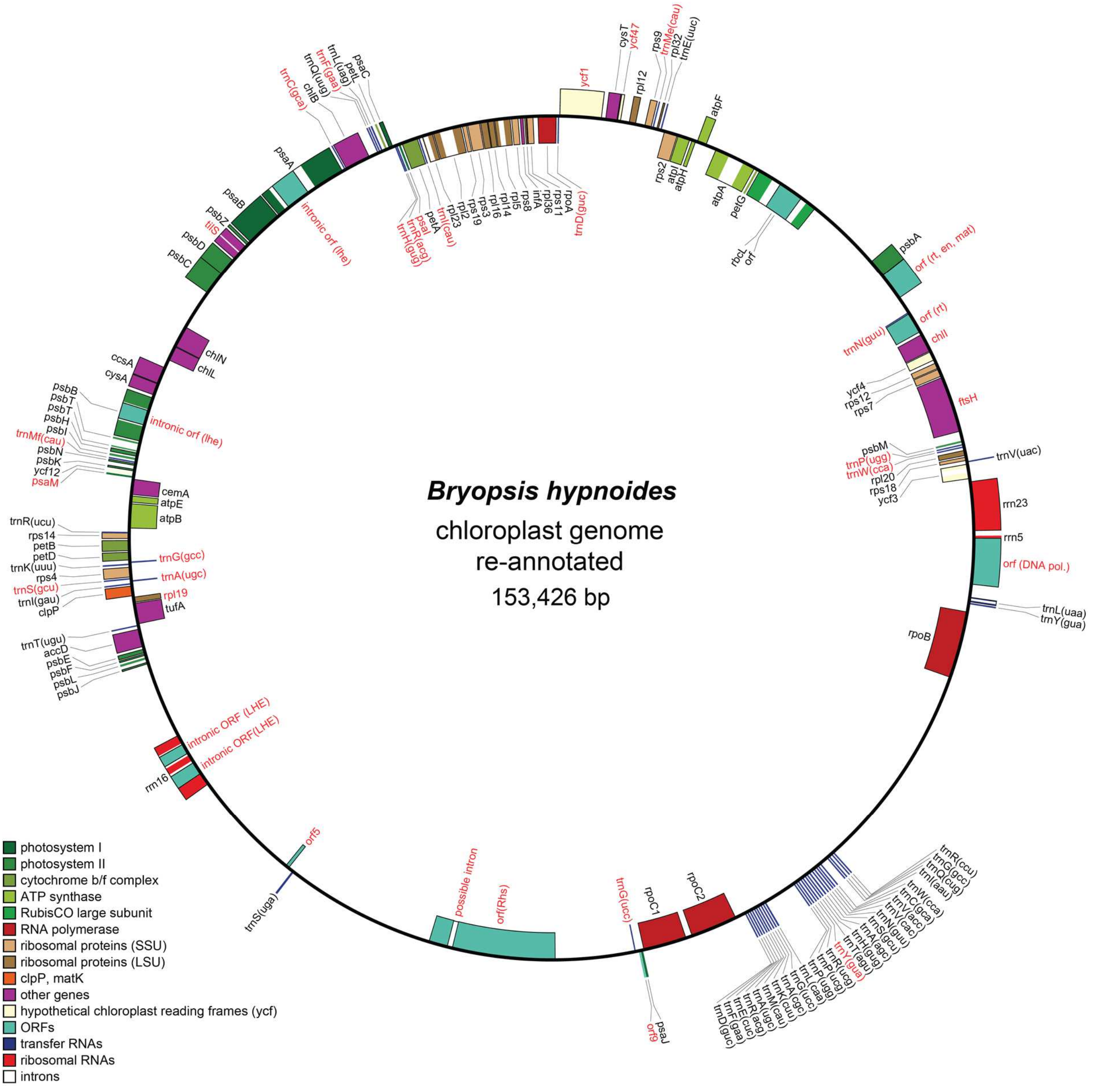

Supplement: Additional file 2: — Re-annotated Bryopsis hypnoides cpDNA. [file 12864_2015_1418_MOESM2_ESM.pdf]
