# Supplementary material for: The chloroplast genomes of Bryopsis plumosa and Tydemania expeditiones (Bryopsidales, Chlorophyta): compact genomes and genes of bacterial origin
Source: BMC Genomics. 2015 Mar 17;16(1):204. doi: 10.1186/s12864-015-1418-3 (PMC4487195; doi:10.1186/s12864-015-1418-3)
Supplement: Additional file 3: — Amino acid alignment of psbM , rpl19 and rpl23 and corresponding trees. [file 12864_2015_1418_MOESM3_ESM.pdf]

Additional file 3. Amino acid alignment of *psbM*, *rpl19* and *rpl23* and corresponding trees.

Amino acid sequences were aligned using the ClustalW translational alignment and a BLOSUM cost matrix, and gap open penalty 10 and gap extend cost 0.1. Phylogenies were estimated under maximum likelihood using RAxML v7.2.7 and the PROTCATDAYHOFF model of amino acid substitution. *Bryopsis* spp. and *Tydemania expeditiones* are indicated in red. The divergent nature of the *Tydemania psbM* sequence as seen in the alignment, resulted in a long branch in the phylogeny.

psbM

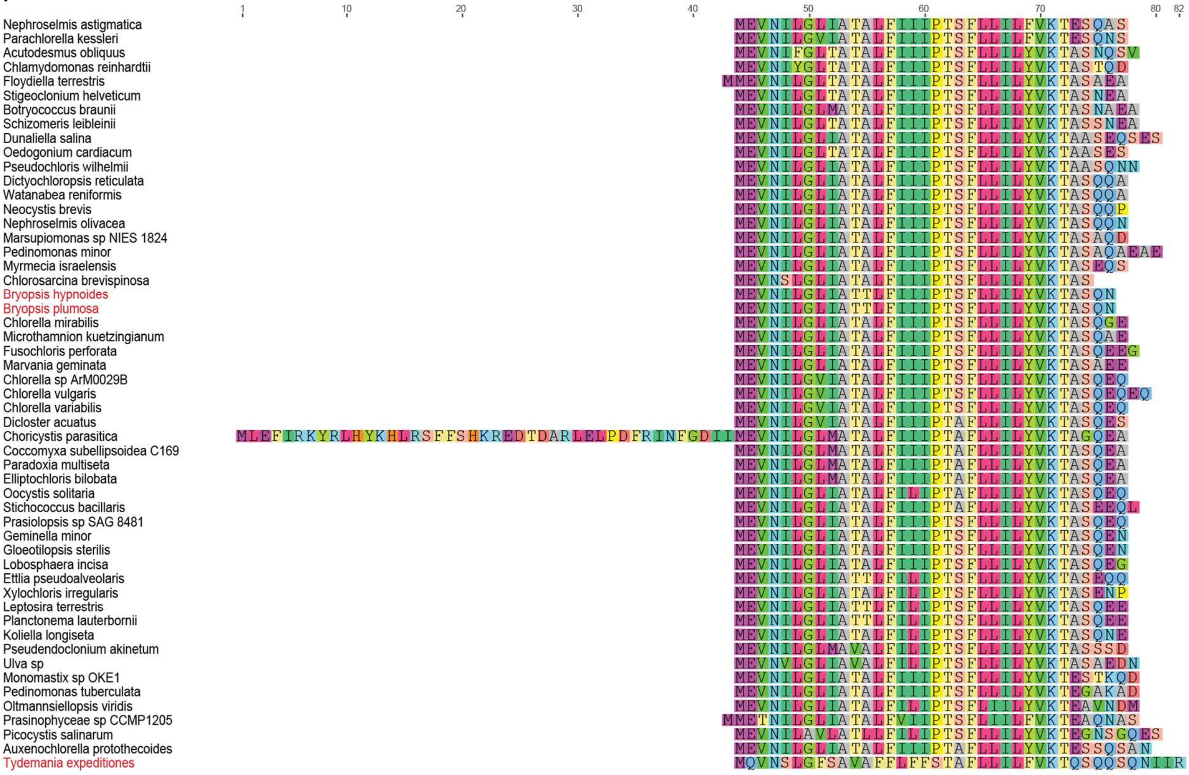

rpl19

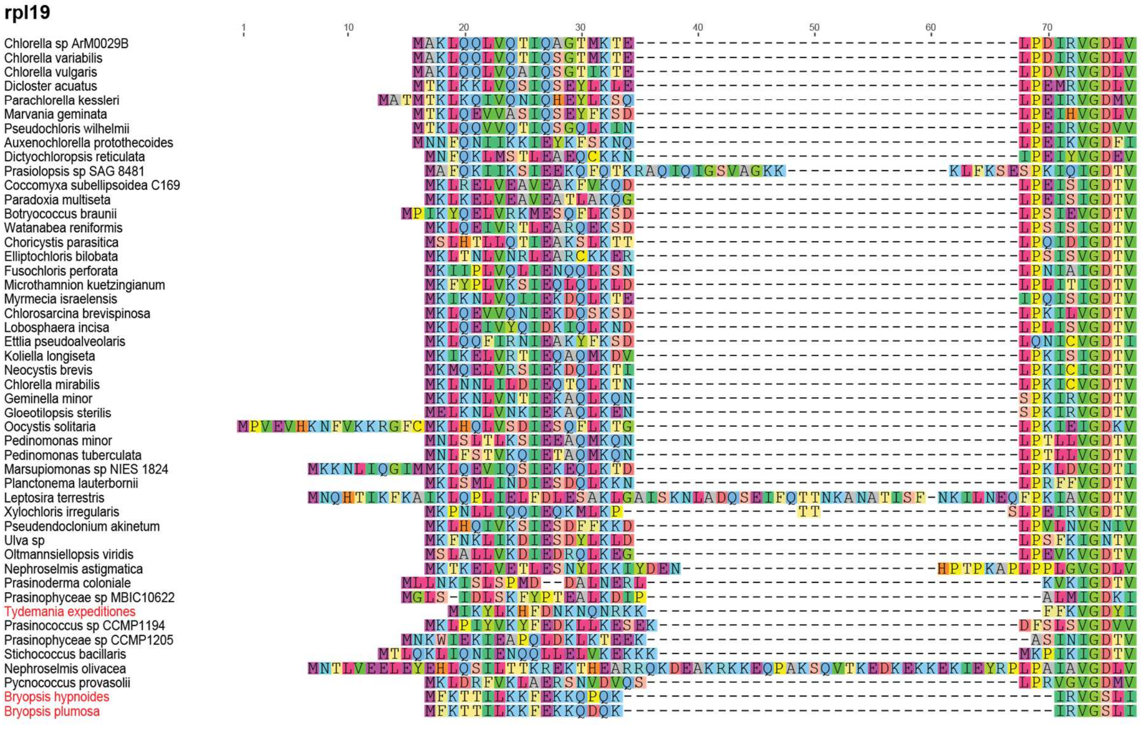

rpl19 (continued)

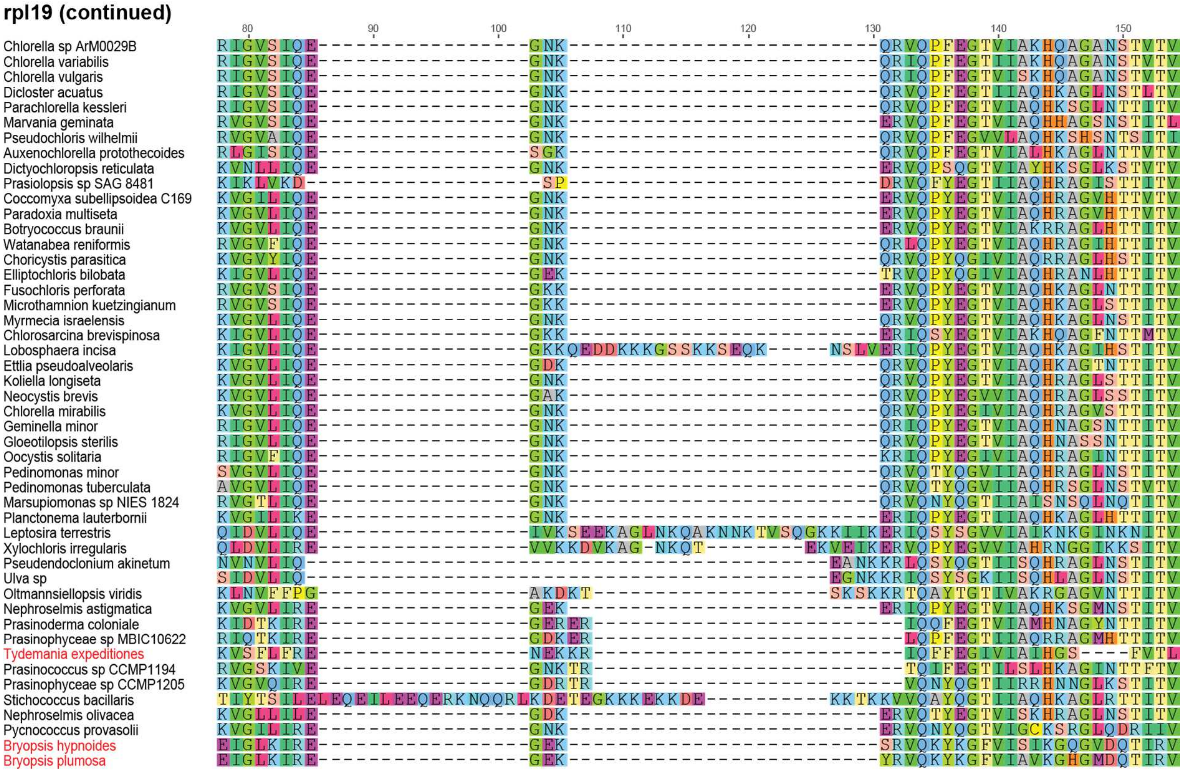

rpl19 (continued)

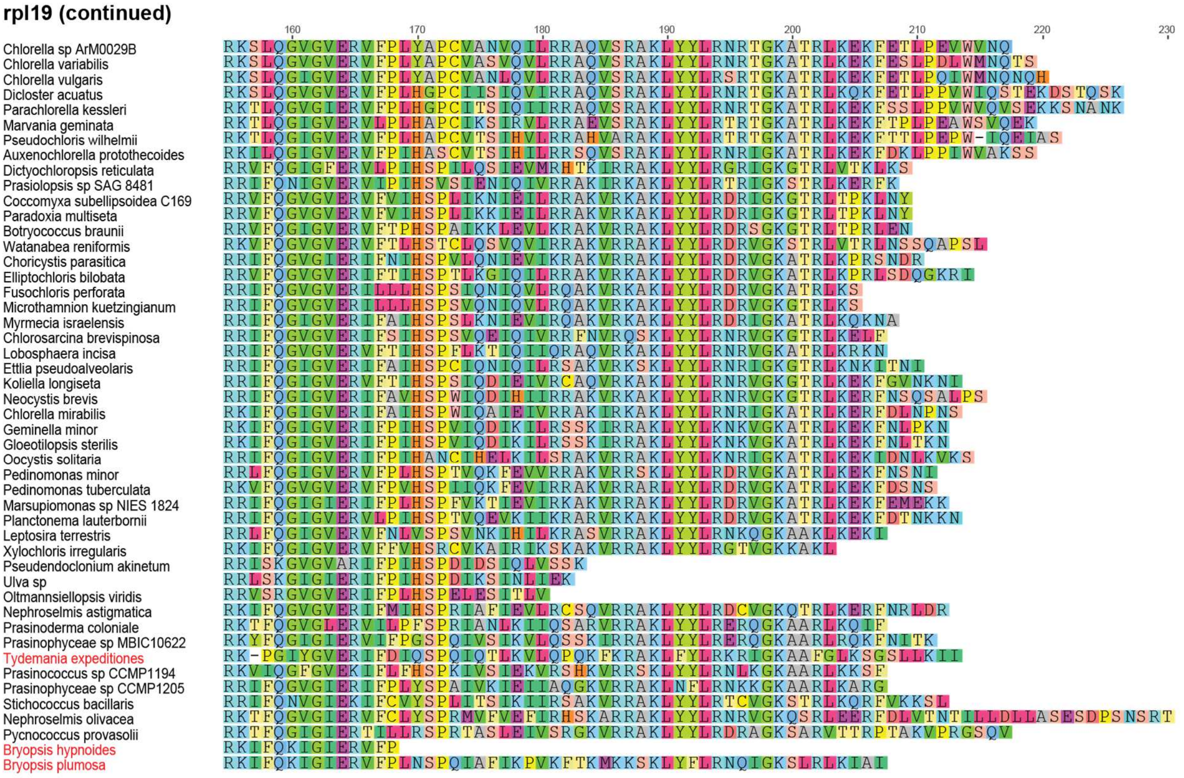

rpl23

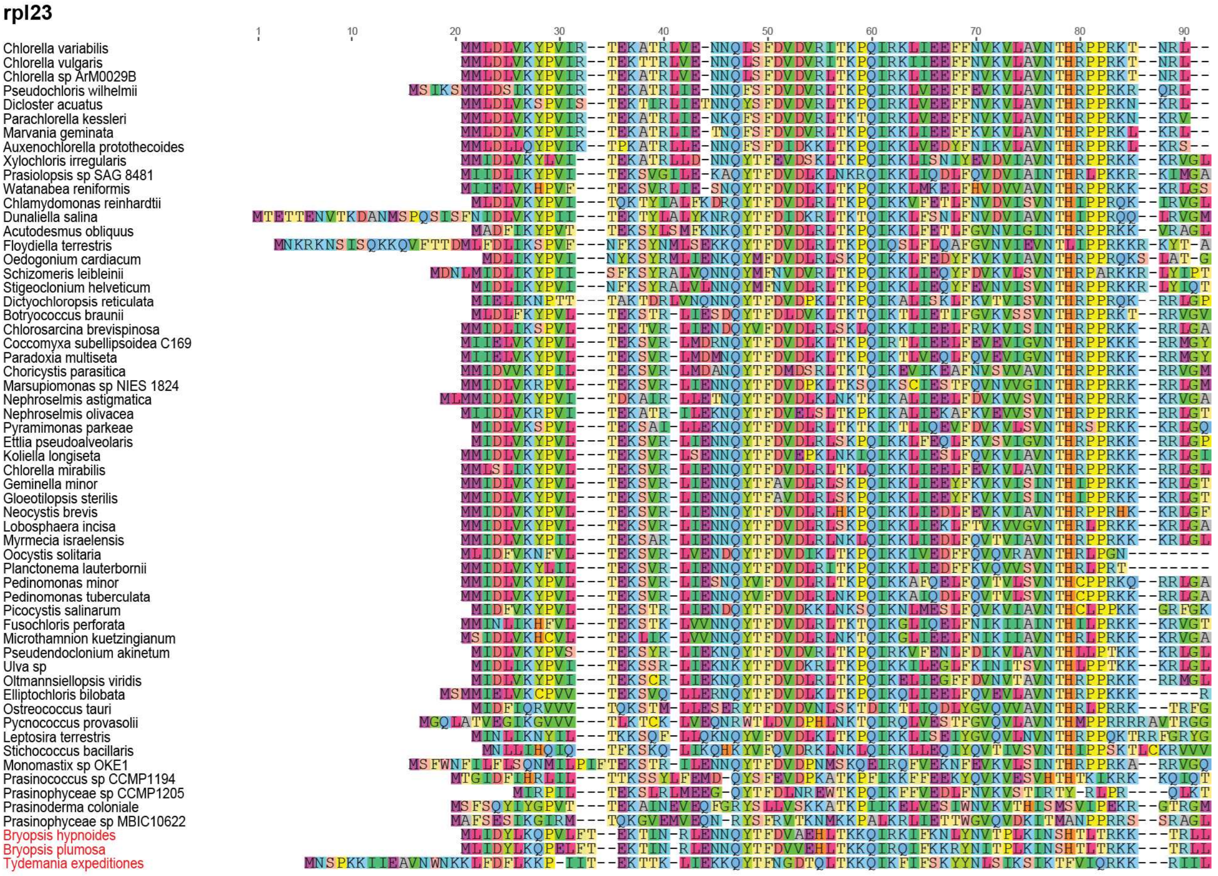

rpl23 (continued)

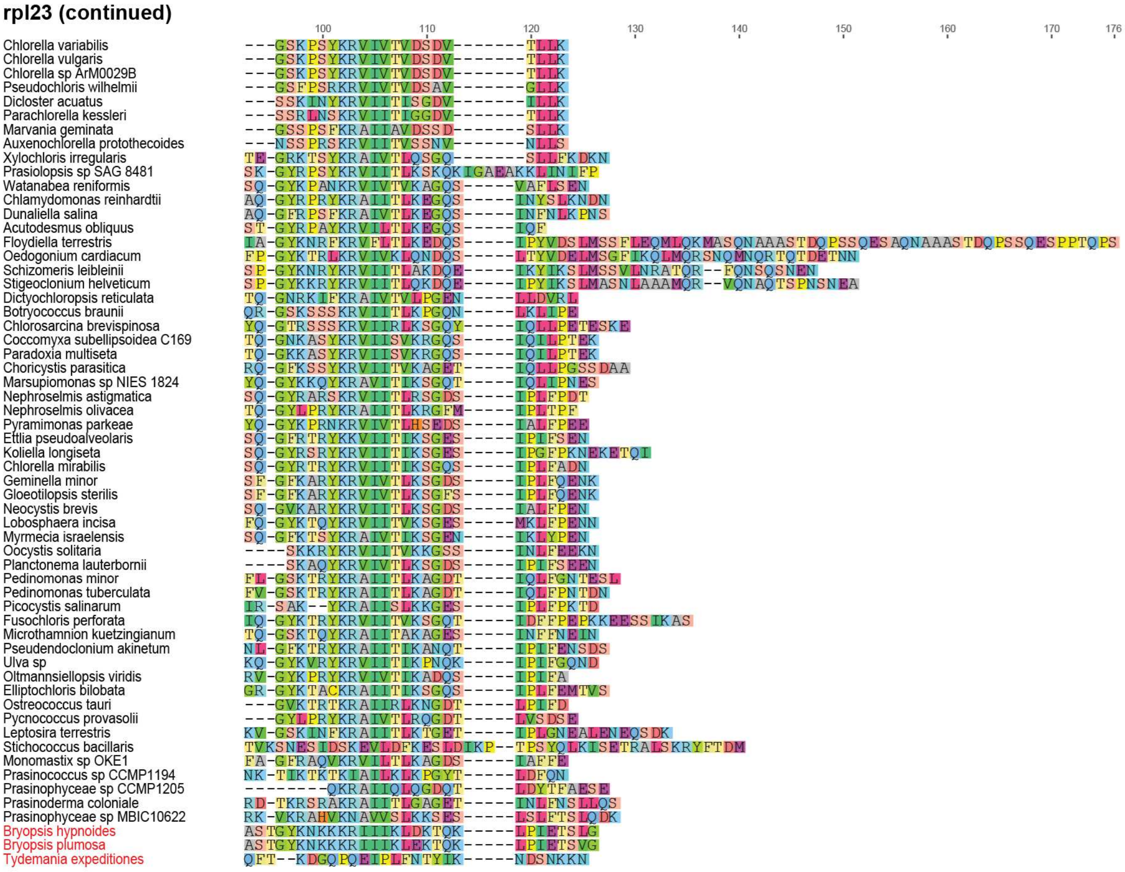

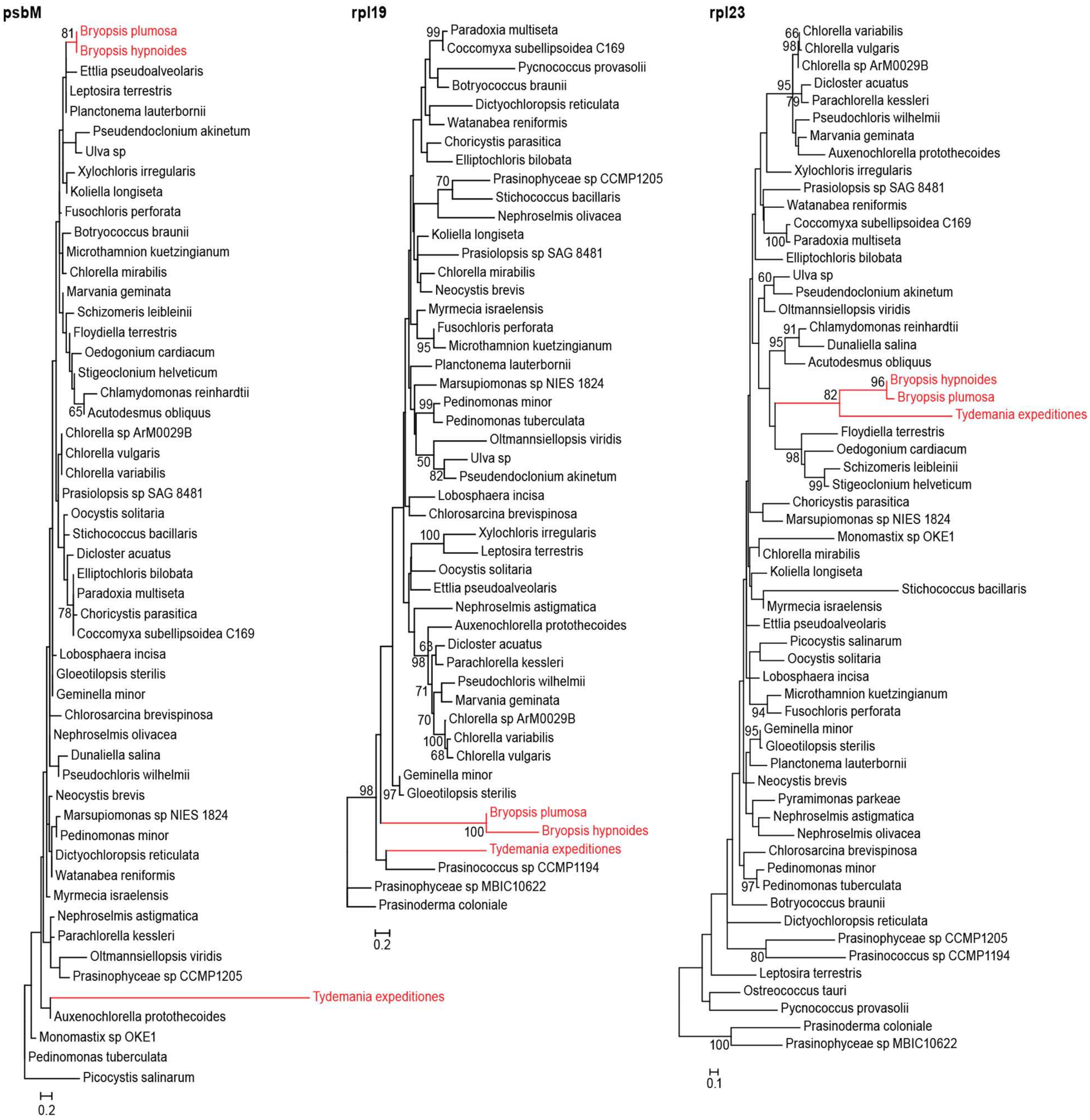
psbM
81 Bryopsis plumosa
Bryopsis hypnoides
Ettlia pseudoalveolaris
Leptosira terrestris
Planctonema lauterbornii
Pseudendoclonium akinetum
Ulva sp
Xylochloris irregularis
Koliella longiseta
Fusochloris perforata
Botryococcus braunii
Microthamnion kuetzingianum
Chlorella mirabilis
Marvania geminata
Schizomeris leibleinii
Floydiella terrestris
Oedogonium cardiacum
Stigeoclonium helveticum
Chlamydomonas reinhardtii
65 Acutodesmus obliquus
Chlorella sp ArM0029B
Chlorella vulgaris
Chlorella variabilis
Prasiolopsis sp SAG 8481
Oocystis solitaria
Stichococcus bacillaris
Dicloster acuatus
Elliptochloris bilobata
Paradoxia multiseta
78 Choricystis parasitica
Coccomyxa subellipsoidea C169
Lobosphaera incisa
Gloeotilopsis sterilis
Geminella minor
Chlorosarcina brevispinosa
Nephroselmis olivacea
Dunaliella salina
Pseudochloris wilhelmii
Neocystis brevis
Marsupiomonas sp NIES 1824
Pedinomonas minor
Dictyochloropsis reticulata
Watanabea reniformis
Myrmecia israelensis
Nephroselmis astigmatica
Parachlorella kessleri
Oltmannsiellopsis viridis
Prasinophyceae sp CCMP1205
Tydemania expeditiones
Auxenochlorella protothecoides
Monomastix sp OKE1
Pedinomonas tuberculata
Picocystis salinarum
0.2
rpl19
99 Paradoxia multiseta
Coccomyxa subellipsoidea C169
Pycnococcus provasolii
Botryococcus braunii
Dictyochloropsis reticulata
Watanabea reniformis
Choricystis parasitica
Elliptochloris bilobata
70 Prasinophyceae sp CCMP1205
Stichococcus bacillaris
Nephroselmis olivacea
Koliella longiseta
Prasiolopsis sp SAG 8481
Chlorella mirabilis
Neocystis brevis
Myrmecia israelensis
Fusochloris perforata
95 Microthamnion kuetzingianum
Planctonema lauterbornii
Marsupiomonas sp NIES 1824
99 Pedinomonas minor
Pedinomonas tuberculata
Oltmannsiellopsis viridis
50 Ulva sp
82 Pseudendoclonium akinetum
Lobosphaera incisa
Chlorosarcina brevispinosa
100 Xylochloris irregularis
Leptosira terrestris
Oocystis solitaria
Ettlia pseudoalveolaris
Nephroselmis astigmatica
Auxenochlorella protothecoides
63 Dicloster acuatus
98 Parachlorella kessleri
71 Pseudochloris wilhelmii
Marvania geminata
70 Chlorella sp ArM0029B
100 Chlorella variabilis
68 Chlorella vulgaris
Geminella minor
98 97 Gloeotilopsis sterilis
Bryopsis plumosa
100 Bryopsis hypnoides
Tydemania expeditiones
Prasinococcus sp CCMP1194
Prasinophyceae sp MBIC10622
Prasinoderma coloniale
0.2
rpl23
66 Chlorella variabilis
98 Chlorella vulgaris
Chlorella sp ArM0029B
95 Dicloster acuatus
79 Parachlorella kessleri
Pseudochloris wilhelmii
Marvania geminata
Auxenochlorella protothecoides
Xylochloris irregularis
Prasiolopsis sp SAG 8481
Watanabea reniformis
Coccomyxa subellipsoidea C169
100 Paradoxia multiseta
Elliptochloris bilobata
60 Ulva sp
Pseudendoclonium akinetum
Oltmannsiellopsis viridis
91 Chlamydomonas reinhardtii
95 Dunaliella salina
Acutodesmus obliquus
96 Bryopsis hypnoides
82 Bryopsis plumosa
Tydemania expeditiones
Floydiella terrestris
98 Oedogonium cardiacum
Schizomeris leibleinii
99 Stigeoclonium helveticum
Choricystis parasitica
Marsupiomonas sp NIES 1824
Monomastix sp OKE1
Chlorella mirabilis
Koliella longiseta
Stichococcus bacillaris
Myrmecia israelensis
Ettlia pseudoalveolaris
Picocystis salinarum
Oocystis solitaria
Lobosphaera incisa
Microthamnion kuetzingianum
94 Fusochloris perforata
95 Geminella minor
Gloeotilopsis sterilis
Planctonema lauterbornii
Neocystis brevis
Pyramimonas parkeae
Nephroselmis astigmatica
Nephroselmis olivacea
Chlorosarcina brevispinosa
Pedinomonas minor
97 Pedinomonas tuberculata
Botryococcus braunii
Dictyochloropsis reticulata
Prasinophyceae sp CCMP1205
80 Prasinococcus sp CCMP1194
Leptosira terrestris
Ostreococcus tauri
Pycnococcus provasolii
Prasinoderma coloniale
100 Prasinophyceae sp MBIC10622
0.1
