# Supplementary material for: The chloroplast genomes of Bryopsis plumosa and Tydemania expeditiones (Bryopsidales, Chlorophyta): compact genomes and genes of bacterial origin
Source: BMC Genomics. 2015 Mar 17;16(1):204. doi: 10.1186/s12864-015-1418-3 (PMC4487195; doi:10.1186/s12864-015-1418-3)

Additional file 4. *petL* amino acid alignment and corresponding phylogenetic tree.

Sequences were aligned using the ClustalW translational alignment with a BLOSUM cost matrix, and gap open penalty 10 and gap extend cost 0.1. Phylogenetic tree based on the *petL* amino acid alignment was estimated under maximum likelihood using RAxML v7.2.7 and the PROTCATDAYHOFF model of amino acid substitution. *Bryopsis* spp. and *Tydemania expeditiones* are indicated in red.

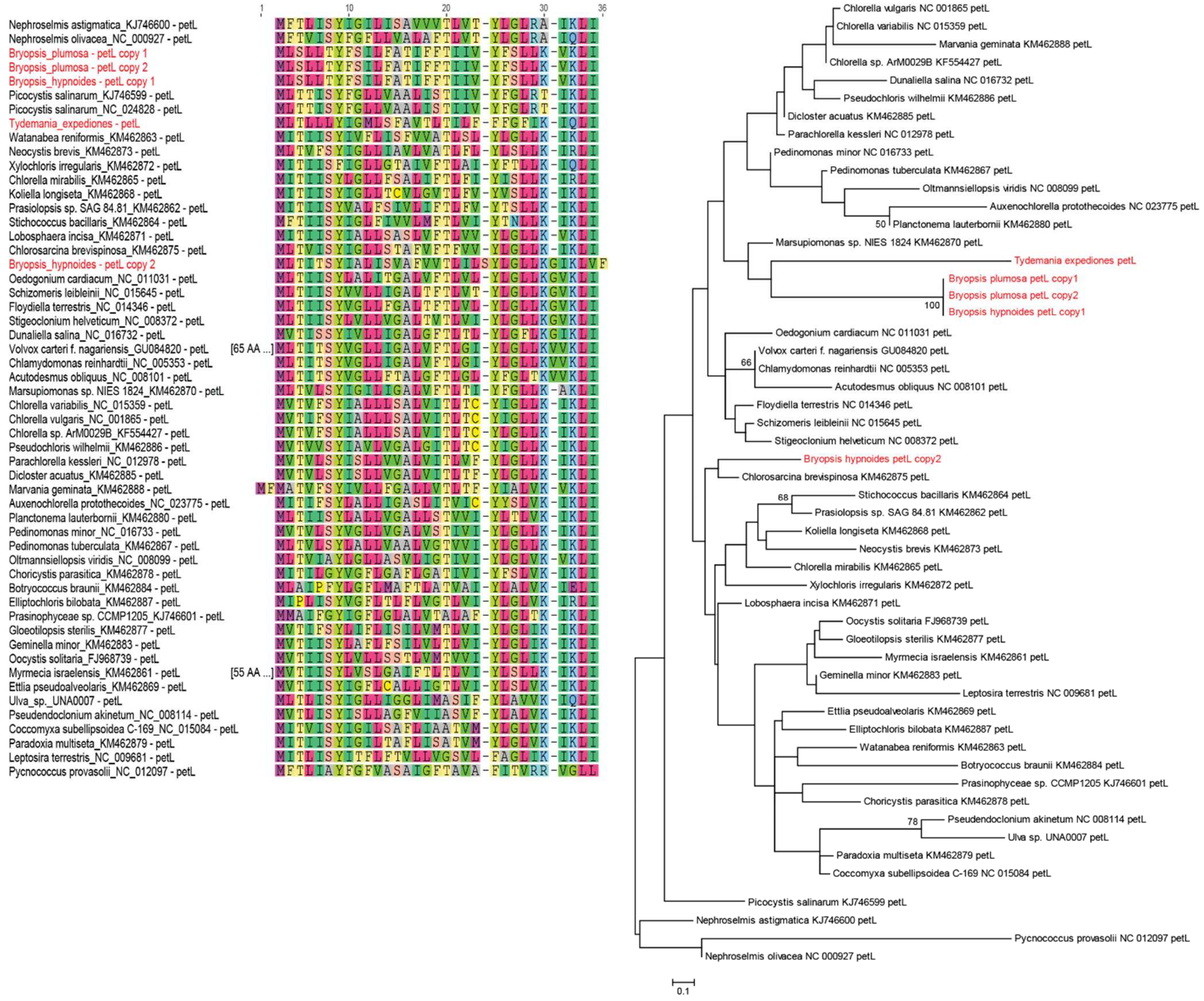

Supplement: Additional file 4: — petL amino acid alignment and corresponding phylogenetic tree. [file 12864_2015_1418_MOESM4_ESM.pdf]
