# Supplementary material for: The chloroplast genomes of Bryopsis plumosa and Tydemania expeditiones (Bryopsidales, Chlorophyta): compact genomes and genes of bacterial origin
Source: BMC Genomics. 2015 Mar 17;16(1):204. doi: 10.1186/s12864-015-1418-3 (PMC4487195; doi:10.1186/s12864-015-1418-3)
Supplement: Additional file 5: — Putative RNA secondary structure of atp A type II intron of Bryopsis plumosa. [file 12864_2015_1418_MOESM5_ESM.pdf]

Additional file 5. Putative RNA secondary structure of *atp*A type II intron of *Bryopsis plumosa*. The RNA structure was modeled according to Michel et al. [1]. Exon sequences are shown in lowercase letters. Roman numbers specify the six major structural domains. EBS and IBS are exon- and intron-binding sites, respectively. The asterisk (*) indicates the site of lariat formation (bulged A in helix VI). Some conserved nucleotides found in type II introns such as the conserved AGC and bulged AC in helix V, the GUGUG motif at the 5'-end, and the AC motif at the 3'-end of the intron are highlighted.

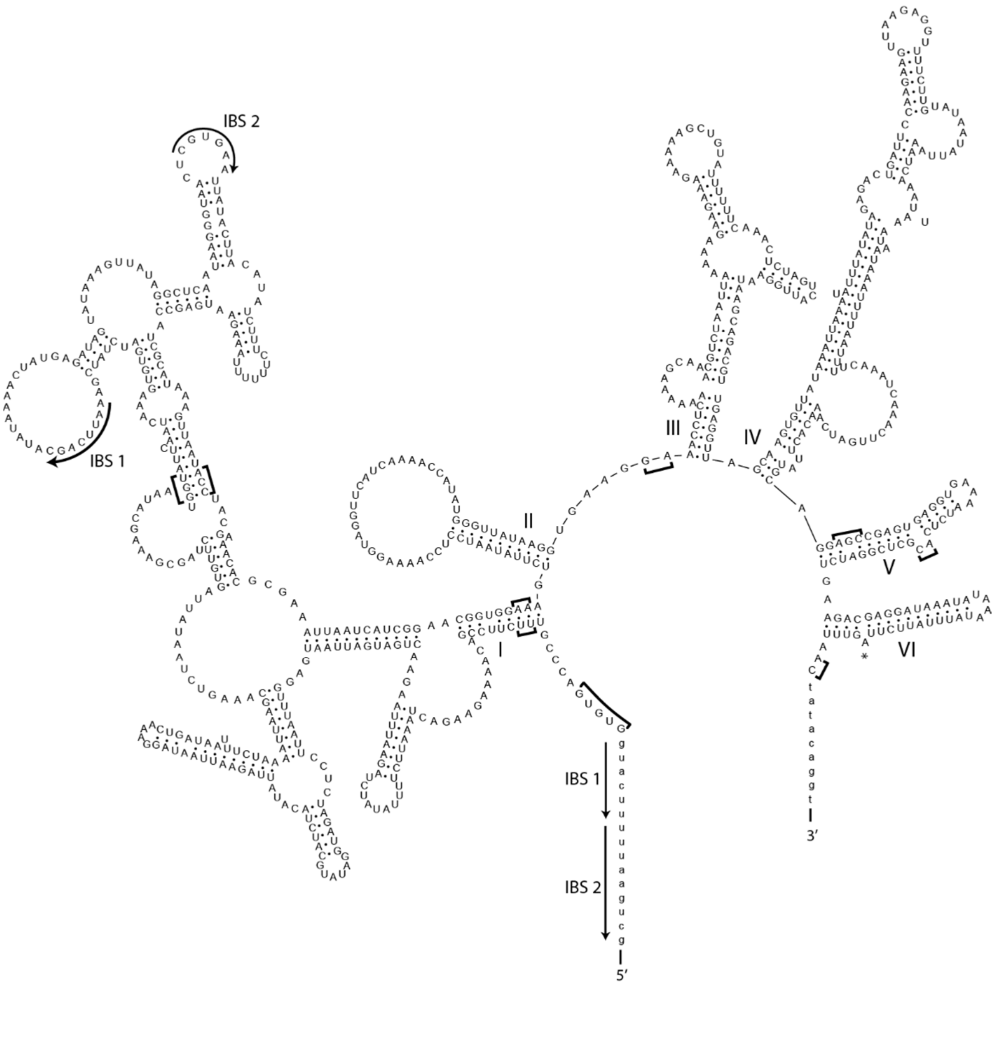

1. Michel F, Umesono K, Ozeki H: **Comparative and functional anatomy of group II catalytic introns - a review**. *Gene* 1989, **82**(1):5-30.
