# Supplementary material for: The chloroplast genomes of Bryopsis plumosa and Tydemania expeditiones (Bryopsidales, Chlorophyta): compact genomes and genes of bacterial origin
Source: BMC Genomics. 2015 Mar 17;16(1):204. doi: 10.1186/s12864-015-1418-3 (PMC4487195; doi:10.1186/s12864-015-1418-3)
Supplement: Additional file 6: — Taxon sampling for the 79-gene and 50-gene datasets. [file 12864_2015_1418_MOESM6_ESM.pdf]

Additional file 6. Taxon sampling for the 79-gene and 50-gene datasets used in the phylogenomic analyses.

**Species**	**79-gene dataset**	**50-gene dataset**	**Genbank accession number(s)**
Acetabularia acetabulum		✓	HG518426-HG518474
Acutodesmus obliquus	✓	✓	NC_008101
Botryococcus braunii	✓	✓	KM462884
Bryopsis hypnoides	✓	✓	NC_013359
Bryopsis plumosa	✓	✓	LN810504
Cephaleuros parasiticus		✓	KM464687, KM464693, KM464698, KM464707, KM464711, KM464715, KM491797, KM491799, KM491808, KM491813, KM491819, KM491823, KM491825, KM491828, KM491837, KM491848, KM504519
Chlamydomonas reinhardtii	✓	✓	NC_005353
Chlorella vulgaris	✓	✓	NC_001865
Coccomyxa subellipsoidea C-169	✓	✓	NC_015084
Dunaliella salina	✓	✓	NC_016732
Elliptochloris bilobata	✓	✓	KM462887
Floydiella terrestris	✓	✓	NC_014346
Geminella minor	✓	✓	KM462883
Halimeda cylindracea		✓	KM820107-KM820166
Koliella longiseta	✓	✓	KM462868
Leptosira terrestris	✓	✓	NC_009681
Lobosphaera incisa	✓	✓	KM462871
Marsupiomonas sp. NIES 1824	✓	✓	KM462870
Marvania geminata	✓	✓	KM462888
Microthamnion kuetzingianum	✓	✓	KM462876
Monomastix sp. OKE-1	✓	✓	NC_012101
Myrmecia israelensis	✓		KM462861
Neocystis brevis	✓	✓	KM462873
Nephroselmis astigmatica	✓	✓	KJ746600
Nephroselmis olivacea	✓	✓	NC_000927
Oedogonium cardiacum	✓	✓	NC_011031
Oltmannsiellopsis viridis	✓	✓	NC_008099
Oocystis solitaria	✓	✓	FJ968739
Ostreococcus tauri	✓	✓	NC_008289
Parachlorella kessleri	✓	✓	NC_012978
Pedinomonas minor	✓	✓	NC_016733
Pedinomonas tuberculata	✓	✓	KM462867
Picocystis salinarum	✓	✓	KJ746599
Prasinococcus sp. CCMP1194	✓	✓	KJ746597
Prasinoderma coloniale	✓	✓	NC_024817
Prasinophyceae sp. CCMP1205	✓	✓	KJ746601
Prasinophyceae sp. MBIC10622	✓	✓	KJ746602
Prasiolopsis sp. SAG 84.81	✓	✓	KM462862
Pseudendoclonium akinetum	✓	✓	NC_008114
Pseudochloris wilhelmii	✓	✓	KM462886
Pycnococcus provasolii	✓	✓	NC_012097
Pyramimonas parkeae	✓	✓	NC_012099
Schizomeris leibleinii		✓	NC_015645
Stigeoclonium helveticum	✓	✓	NC_008372
Tetraselmis spp		✓	AB561008-AB561081, DQ173248, DQ173249, DQ227304, HF931099
Trebouxia aggregata		✓	EU123963-EU124002
Trentepohlia annulata		✓	KM464689, KM464692, KM464697, KM464705, KM464706, KM464712, KM464717, KM491796, KM491802, KM491811, KM491820, KM491824, KM491826, KM491831, KM491834, KM491839, KM491841, KM491845
Tydemania expeditiones	✓	✓	LN810505
Ulva sp. UNA00071828	✓	✓	KP720616
Volvox carteri		✓	GU084820
Watanabea reniformis	✓	✓	KM462863
Xylochloris irregularis	✓	✓	KM462872
